# Why Do You Believe in God? Relationships between Religious Belief, Analytic Thinking, Mentalizing and Moral Concern

**DOI:** 10.1371/journal.pone.0149989

**Published:** 2016-03-23

**Authors:** Anthony Ian Jack, Jared Parker Friedman, Richard Eleftherios Boyatzis, Scott Nolan Taylor

**Affiliations:** 1 Department of Philosophy, College of Arts & Sciences, Case Western Reserve University, Cleveland, Ohio, United States of America; 2 Inamori International Center for Ethics and Excellence, Case Western Reserve University, Cleveland, Ohio, United States of America; 3 Department of Organizational Behavior, Weatherhead School of Management, Case Western Reserve University, Cleveland, Ohio, United States of America; 4 Department of Psychology, College of Arts & Sciences, Case Western Reserve University, Cleveland, Ohio, United States of America; 5 Department of Neurology, Medical School, Case Western Reserve University, Cleveland, Ohio, United States of America; 6 Department of Neurosciences, Medical School, Case Western Reserve University, Cleveland, Ohio, United States of America; 7 Management Division, Babson College, Babson Park, Massachusetts, United States of America; University of L'Aquila, ITALY

## Abstract

Prior work has established that analytic thinking is associated with disbelief in God, whereas religious and spiritual beliefs have been positively linked to social and emotional cognition. However, social and emotional cognition can be subdivided into a number of distinct dimensions, and some work suggests that analytic thinking is in tension with some aspects of social-emotional cognition. This leaves open two questions. First, is belief linked to social and emotional cognition in general, or a specific dimension in particular? Second, does the negative relationship between belief and analytic thinking still hold after relationships with social and emotional cognition are taken into account? We report eight hypothesis-driven studies which examine these questions. These studies are guided by a theoretical model which focuses on the distinct social and emotional processing deficits associated with autism spectrum disorders (mentalizing) and psychopathy (moral concern). To our knowledge no other study has investigated both of these dimensions of social and emotion cognition alongside analytic thinking. We find that religious belief is robustly positively associated with moral concern (4 measures), and that at least part of the negative association between belief and analytic thinking (2 measures) can be explained by a negative correlation between moral concern and analytic thinking. Using nine different measures of mentalizing, we found no evidence of a relationship between mentalizing and religious or spiritual belief. These findings challenge the theoretical view that religious and spiritual beliefs are linked to the perception of agency, and suggest that gender differences in religious belief can be explained by differences in moral concern. These findings are consistent with the opposing domains hypothesis, according to which brain areas associated with moral concern and analytic thinking are in tension.

## Introduction

In the last decade, a number of theorists and experimentalists have attempted to address the question of what psychological mechanisms give rise to religious and spiritual beliefs. Two major themes have emerged, one examining the relationship with analytic reasoning skills, and a second examining the relationship with social cognition. There is mounting evidence, both correlational and causal, which demonstrates that analytic thinking (as measured by tests of intelligence and critical thinking) discourages the acceptance of religious and spiritual beliefs [1–7]. One interpretation of these findings is that analytic thinking decreases belief because it encourages individuals to carefully evaluate data and arguments and/or override certain intuitively appealing beliefs [2, 4, 6].

Whereas analytic thinking discourages religious and spiritual beliefs, there is both theoretical and empirical support for the view that belief is positively linked to social cognition. A number of theorists have argued that the tendency to perceive agency and intentionality encourage belief in supernatural agents. Broadly supporting this view, a number of studies and theories have linked measures of empathy, social cognition, and emotion self-regulation—including measures of social and emotional intelligence—to religious and spiritual belief [8–22]. However, it is now recognized that social cognition can be subdivided into a number of distinct dimensions, both behaviorally and neurologically [23–26]. Hence, it remains to be established which specific dimensions of social cognition are most strongly linked to religious belief.

Analytic thinking and social cognition not only have opposing effects on religious and spiritual belief, but it has also been hypothesized that these types of thinking may be in competition [24, 27–31]. For example, there is evidence that males tend to be stronger at systemizing, whereas females tend to be stronger at empathizing, and that high functioning individuals with autism spectrum disorder (ASD) represent an extreme form of imbalance between these two types of thinking [e.g., an extreme form of the male brain; 32]). If there is competition between analytic thinking and social cognition, then analytic reasoning may be negatively correlated with certain dimensions of social cognition across individuals. Hence, the negative relationship between analytic thinking and religious/spiritual belief might be explained by two factors: A positive association between an aspect of social cognition and religious belief; and competition between that aspect of social cognition and analytic thinking. It follows that it is important to establish the strength of the relationship between religious/spiritual belief and measures of social cognition and analytic thinking after taking into account relationships between those measures.

We have previously advanced a theoretical account of the mind-body problem inspired by the observation that ASD and psychopathy are associated with distinct deficits in social cognition [24, 33–35]. While the ASD phenotype is associated with a variety of behavioral manifestations, numerous researchers identify the primary characteristic of ASD as a deficit in mentalizing (e.g. theory mind and behavior decoding tasks; [32, 33, 35, 36]). The psychopathic phenotype is also associated with a variety of personality characteristics, including impulsivity and aggression, however the primary personality characteristic of psychopathy has been identified as callous affect—an absence of emotional response to pain and suffering in others [37]. Psychopathy has also been associated with deficits in interpersonal connection, prosocial behavior and moral reasoning [33, 34, 37].

There is evidence that the deficit in mentalizing associated with ASD and the trait of callous affect seen in psychopathy are distinct. Individuals with psychopathy have normal or better than normal mentalizing abilities [38], a feature which helps explain their ability to manipulate others. Conversely, the ASD phenotype is not generally associated with callous affect, i.e., a deficit in feelings of empathic concern for others [39–41]. The clinical dissociation between ASD and psychopathy is also reflected by a dissociation in the associated traits which is evident in the non-clinical population [33, 42].

We broadly characterize the social deficit associated with psychopathy as a deficit in moral concern [24]. The concept of moral concern is motivated by a basic distinction in moral philosophy between moral patiency and moral agency, which are associated with distinct moral sentiments of concern and blame, respectively [43]. Work in psychology also supports the view that perceptions of moral patiency are dissociable from (and sometimes in tension with) perceptions of moral agency [44–46]. In addition, we have shown that certain moral judgment tasks which hinge on perceptions of moral patiency are impacted by personality characteristics associated with psychopathy (i.e., deficits in empathic concern) but not by individual differences in mentalizing (i.e., deficits in theory of mind) [31]. Based on a review of this work, we identify moral concern as a broad category which includes empathic concern, interpersonal connection, prosocial behavior and aspects of moral reasoning. It is important to note that individuals with high levels of moral concern will not necessarily behave more ethically in all situations. Indeed, some researchers have claimed that high levels of empathic concern can be a detriment to moral behavior [47]. Further, there is empirical support for the view that moral concern for others can lead to aggression in the context of perceived threat [48]

Our theory, the opposing domains hypothesis [24, 29, 30], holds that our neural architecture has evolved in such a way that it creates a tension between analytic thinking and moral concern. This contrasts with Baron-Cohen’s model, which emphasizes a tension between analytic thinking and aspects of social cognition impacted by ASD (i.e. mentalizing). Empirical support for our theory derives primarily from work in neuroimaging. First, reviews of the neuroscience literature, including formal meta-analyses, support the view that these two broad domains (analytic thinking and moral concern) map onto two anatomically discrete cortical networks. The task positive network (TPN) is consistently activated by cognitively demanding non-social tasks, including mathematical, physical and logical reasoning tasks [29, 49–53]. Individual differences in these skills are associated with increased TPN activation during these tasks [52, 54]. The default mode network (DMN) is consistently activated by social and emotional cognition [55, 56]. Our hypothesized broad cognitive category of ‘moral concern’, suggested to us by the personality profile of individuals with psychopathy, maps well onto the known functions of the DMN. Greater activity in the DMN has been associated with more empathic concern [57–59], social connection (i.e. reverse of prejudice and disconnection) [30, 60–65], prosocial behavior [60, 66, 67] and moral reasoning [68–70]. Notably many of these studies link individual differences in these characteristics to DMN activity [57, 59, 60, 66, 67].

Second, it has long been known that the TPN and DMN exhibit an antagonistic relationship, in the sense that activation of one network corresponds with deactivation of the other network below resting baseline. Initially, it was observed that a broad range of cognitively demanding non-social tasks (which we characterize broadly as involving ‘analytic reasoning’) not only activate the TPN but also deactivate the DMN [50, 71]. It was later found that the TPN and DMN also tend to be in tension during ‘spontaneous cognition’, i.e. when the participant is not given any task [72]. This phenomenon is referred to as ‘resting anti-correlation’ between the networks. It suggests that competition between the networks is an emergent property of the network architecture of the brain. Finally, we have demonstrated that attention to engaging social stimuli not only activates the DMN but also deactivates the TPN. In a subsequent study[30] it was shown that this pattern of DMN activation and TPN deactivation was present for humanizing depictions of individuals, whereas dehumanizing depictions, which are associated with decreased moral concern, either involved decreased activity in the DMN or increased activity in the TPN. Taken together, these findings suggest that we are neurologically constrained from simultaneously exercising moral concern and analytic thinking.

We suggest that this structural feature of the brain underlies the long noted anecdotal tension between materialistic and spiritual worldviews. This linkage is supported by three observations. First, brain areas implicated in analytic thinking (TPN) support cognitive process essential for maintaining a naturalistic world view (e.g. thinking about objects, mechanisms and causes; [29, 49, 71, 73–77]), whereas the brain areas implicated in moral concern (DMN) are associated with thinking about phenomena which have traditionally been thought of as non-physical, namely minds and emotions [78–83]. Second, brain areas associated with materialism (TPN) tend to be suppressed when brain areas associated with moral concern (DMN) are activated [29, 71, 72]. This might explain the tendency to link mind with spirit, i.e. the view that minds and emotions are associated with the extra- or super- natural. Third, brain areas associated with analytic thinking are associated with religious disbelief [73, 74, 84], and brain areas associated with moral concern are associated with religious belief [73] and prayer [84, 85].

### The Present Research

In the studies reported here, we examine the relationship between belief in God and/or a universal spirit and individual difference measures that characterize the domains of cognition of theoretical interest (in particular analytic thinking and moral concern). Numerous findings cited above support the view that increased activity in the TPN and DMN is associated with individual differences in the relevant cognitive domains. However, it is important to note that these findings do not necessitate a negative correlation between individual difference measures of analytic reasoning and moral concern. The neuroimaging findings show there is a constraint on activating both brain networks at the same time. However, tests of empathic concern and of analytic thinking measure people’s ability within specific contexts. Engaging social stimuli are associated with activation of the DMN and deactivation of the TPN, whereas analytic problems are associated with activation of the TPN and deactivation of the DMN. Hence, there is no contradiction inherent in an individual excelling in both domains, provided they engage and disengage the DMN and TPN in a manner appropriate to the context. Indeed, it is plausible that this feature of the brain’s organization is present precisely so that analytic and empathetic thinking do not interfere with each other.

The tension between the brain networks is hypothesized to be behaviorally relevant when people are faced with ambiguous or mixed stimuli, which participants might respond to either by engaging analytic thinking or by engaging empathy. Our claim is that in these ambiguous cases, the balance of the individual’s abilities/tendencies will determine how likely they are to respond by engaging one network rather than the other. Our working hypothesis is that religious and spiritual stimuli provide such an ambiguous context.

Three hypotheses concerning the relationship between different measures of social and non-social thinking follow directly from the opposing domains model: (i) individual difference measures of analytic thinking and intelligence negatively predict belief in God or a universal spirit; (ii) even controlling for the link to analytic reasoning, self and peer-report measures of moral concern positively predict belief; (iii) individual difference measures of mentalizing are not significantly positively related to belief after controlling for links to analytic thinking and moral concern. To our knowledge, no single set of studies has simultaneously examined the relationship these three constructs share with religious and spiritual beliefs.

Studies 1–3 examine the opposing effects of measures of analytic thinking and moral concern on religious and spiritual belief. Studies 4–7 demonstrate that once the predictive effects associated with measures of analytic thinking and moral concern are taken into account, measures which either directly or indirectly assess mentalizing do not add any predictive power in the regression model. Study 8 demonstrates that the positive relationship between moral concern and belief is not due to socially desirable responding or the effects of social contact due to religious affiliation.

### Ethics Statement

All studies were approved by the Institutional Review Board (IRB) at Case Western Reserve University. All participants provided consent to participate in the studies and were financially compensated. The financial compensation for participants in each study, detailed account of the procedures, and correlation and regression tables can be found in S1 Supporting Information. Attention check questions were included in all of the Amazon Mechanical Turk (AMT) studies. The supporting data sets for each study can be found in S1 Data.

Written consent was provided by the participants in Study 5. All other studies did not acquire consent, as these studies all employed Amazon Mechanical Turk, where the minimum age to register is 18 and no personally identifiable information is made available or linked to the participant’s responses. These procedures were approved by the IRB.

## Results and Discussion

### Study 1

Study 1 examined a sample of 236 adults from the USA (recruited from Amazon’s Mechanical Turk; AMT) to test our hypothesis that religious and spiritual beliefs reflects a tension between moral concern and analytic thinking. Moral concern was indexed with the 7-item Empathic Concern subscale of the IRI ([26]). A sample item from the IRI-EC is “I often have tender, concerned feelings for people less fortunate than me” One measure assessed critical reasoning (Cognitive Reflection Test; CRT) [86] and the other mechanical reasoning (Intuitive Physics Test; IPT) [28]. These two measures fall under the broader category of analytic thinking. A sample item of the CRT is “If it takes 5 machines 5 minutes to make 5 widgets, how long would it take 100 machines to make 100 widgets”. For this and all remaining studies, the calculated CRT score reflects the proportion of questions the participant answered correctly (the correct answer to the example item above is 5). The IPT requires diagrams, so we cannot include a sample item. To measure religious and spiritual beliefs we used the following single item measure in this and all following studies: “Do you believe in the existence of either God or a universal spirit?” This question was answered on a 7-point Likert scale (1 = *not at all*; 7 = *definitely yes*). The wording of this item allows individuals to express the degree to which they believe in any God, regardless of the religious system, and/or belief in a universal spirit, which tends to be associated with spirituality. These are concepts which largely overlap, but do have important differences [87]. What is important for the present set of experiments is that both concepts are non-materialistic and are both associated with moral concern [8–22].

Participants completed all measures in the following order: demographics and belief, IPT, IRI-EC, and CRT.

Correlational analyses showed that belief was positively correlated with IRI-EC (*r* = 0.30, *p*<.001) and negatively correlated with the CRT (*r* = -0.15, *p*<.05) and IPT (*r* = -0.13, *p*<.05). The IRI-EC was negatively correlated with the CRT (*r* = -0.17, *p*<.01) and IPT (*r* = -0.18, *p*<.01).

We entered all variables into a 3-step hierarchical linear regression analysis predicting belief. From here forward, we report the significance of each model, and only include the regression coefficients for all study variables as they appear in the final step. comprehensive regression table for each study can be found in S1 Data. Entering gender, age and education in the first step produced a statistically significant modelΔR^2^ = .05, Δ*F* (3, 232) = 3.96, *p* < .01). We use Cohen’s f^2^ [88] to report the effect size (Cohen’s f^2^ small = 0.02, medium = 0.15, large = 0.35 of each additional step in the regression model. According to Cohen’s f^2^ the effect size of adding the demographic variables was small (0.052). Adding IRI-EC to the second step significantly improved the model (ΔR^2^ = .063, Δ*F* (1, 232) = 16.381, *p* < .001) and produced a small effect size (Cohen’s f^2^ = 0.07). Adding CRT and IPT in the third step failed to produce a statistically significant better model than the previous (ΔR^2^ = .014, Δ*F* (2, 229) = 1.804, *p* = .17) and failed to produce a small effect size (Cohen’s f^2^ = 0.014). In the final step, religious belief was only predicted by IRI-EC (*β* = 0.24, *p* < .001) and age (*β* = 0.16, *p* < .05). No other variables were significant: CRT (*β* = -0.09, *p* = .19), IPT (*β* = -0.07, *p* = .33), gender (*β* = 0.01, *p* = .91), education (*β* = -0.03, *p* = .69).

Study 1 suggests that the relationship between analytic thinking and disbelief may be at least partially accounted for by the observed negative correlations between analytic thinking and moral concern, and the positive relationship between empathic concern and belief in God or a universal spirit.

### Study 2

Study 2 aimed to extend the results of Study 1 by including the 16-item Mill-Hill Vocabulary scale, which we used as a measure of crystallized intelligence (CI)[89]. Participants were presented a bolded-word with five words underneath, and asked to “select one of the five definitions that best fits the bold word”. Using a sample of 233 adults from the USA (recruited from AMT), we tested the hypothesis that belief would be positively predicted by empathic concern (IRI-EC) after controlling for any observed negative effect indexed by measures of analytic thinking (CRT and IPT) and crystallized intelligence (CI).

Participants completed all measures in the following order: demographics and belief, IPT, IRI-EC, Mill-Hill vocabulary, and CRT.

Bivariate correlations showed that belief was positively correlated with IRI-EC (*r* = 0.35, *p* <.001) and negatively correlated with the CRT (*r* = -0.25, *p* <.001), the IPT (*r* = -0.16, *p* <.05) and CI (*r* = -0.19, *p* <.01). The IRI-EC was negatively correlated with the CRT (*r* = -0.22 *p* <.001) and IPT (*r* = -0.23, *p* <.001) but not CI (*r* = 0.04, *p* = *ns*).

We entered all variables into a 3-step hierarchical regression analysis predicting belief. Entering gender, age and education into the first step provided a significant model (ΔR^2^ = .036, Δ*F* (3, 229) = 2.837, *p* < .05) and produced a small effect size (Cohens f^2^ = 0.037). Entering IRI-EC into the second step significantly improved the model (ΔR^2^ = .097, Δ*F* (1, 228) = 25.379, *p* < .001) and produced a small effect size (Cohens f^2^ = 0.11). Entering CRT, IPT and CI into the final step significantly improved the model (ΔR^2^ = .061, Δ*F* (3, 225) = 5.631, *p* < .001) and together produced a small effect size (Cohens f^2^ = 0.076). In the final step, belief was positively predicted by empathic concern (*β* = 0.31, *p* < .001) and negatively predicted by CI (*β* = -0.21, *p* < .01). No other variables were significant: CRT (*β* = -0.12, *p* = .08), IPT (*β* = 0.01, *p* = .86), gender (*β* = 0.06, *p* = .32), age (*β* = 0.10, *p* = .12) and education (*β* = 0.01, *p* = .91)

Again, study 2 suggests that measures of analytic thinking that negatively predict belief may do so only because they are negatively correlated with a measure of empathic concern, which is positively associated with belief.

### Study 3

This study recruited a sample of 159 adults from the USA (from AMT) in order to replicate and extend our previous results by including an additional measure of moral concern. We used the Identification With All of Humanity scale to assess the extent to which one feels empathic connection with out-group members [90]. A representative item is “How much do you identify with (that is, feel a part of, feel love toward, have concern for), each of the following”. Participants answered these questions on a 5-point Likert-scale for each of the following groups “people in my community; people in my country; all of humanity”. We only report scores for the “all of humanity” subscale (IWAH_Global), as this is the most relevant to the empirical predictions. We also included self-report measures assessing political stances on social and economic issues [91]. This study only included one measure of analytic thinking (CRT).

Participants completed all measures in the following order: IRI-EC, CRT, IWAH scale, and questions about belief and demographics.

Bivariate correlations showed that belief was positively related to IRI-EC (*r* = 0.28, *p* <.001) and IWAH_Global (*r* = 0.34, *p* <.001). It was negatively related to CRT (*r* = -0.22, *p* <.01). CRT was negatively correlated with IWAH_Global (*r* = -0.24, *p* <.01) but not with IRI-EC (*r* = 0.02, *p = ns*).

We entered all variables into a 4- step hierarchical regression analysis predicting belief. Entering demographics failed to produce a statistically significant model (ΔR^2^ = .009, Δ*F* (3, 155) = .448, *p* = .719) and failed to produce a small effect size (Cohens f^2^ = 0.009). Entering both IRI-EC and IWAH_Global into the second step significantly improved the model (ΔR^2^ = .152, Δ*F* (2, 153) = 13.894, *p* <.001), and together produced a medium effect size (Cohens f^2^ = 0.181). Entering CRT into the third step significantly improved the model (ΔR^2^ = .026, Δ*F* (1, 152) = 4.910, *p* < .05) and produced a small effect size (Cohens f^2^ = 0.032). Entering the questions about social and economic issues significantly improved the model (ΔR^2^ = .037, Δ*F* (2, 150) = 3.608, *p* < .05) and together had a small effect size (Cohens f^2^ = 0.049). The final step of the regression showed that belief was positively predicted by empathic concern (*β* = 0.26, *p*< .01), IWAH_Global (*β* = 0.23, *p*< .01) and leaning conservative on social issues (*β* = 0.26, *p* < .01). No other variables were significant: CRT (*β* = -0.15, *p* = .06), economic issues (*β* = -0.19, *p* = .06) gender (*β* = 0.02, *p* = .84), age, (*β* = -0.06, *p* = .39) and education (*β* = 0.10, *p* < .17).

Study 3 establishes that belief in God or a universal spirit is independently predicted by self-reports of empathic concern and the extent to which one feels concern for all of humanity (IWAH_Global).

Studies 1–3 all suggest religious and spiritual disbelief may no longer be predicted by analytic thinking after its negative relationship with measures of empathy are taken into account.

Studies 4–7 extend these investigations by including direct and indirect measures of mentalizing to test whether they add predictive power when various measures of empathic concern and prosocial behavior are also taken into account.

### Study 4

Study 4 recruited a sample of 527 adults from the USA (from AMT) to test the prediction that, once we control for measures of empathic concern and analytic thinking, a measure of mentalizing does not add any predictive power to the regression model. The mentalizing measure used was the perspective taking subscale of the Interpersonal Reactivity Index (IRI-PT) [26], the same instrument that includes the empathic concern subscale. A representative item is: “I try to look at everybody’s side of a disagreement before I make a decision.” Study 4 also included a 6-item self-report measure of prosocial intentions [92]. This scale asks to participants “to what extent do you intend, in the next six weeks, to… A representative item is “Go out of your way to help a stranger in need.” The CRT was included as a measure of analytic thinking.

Participants completed all measures in the following order: Demographics and belief, CRT, IRI-EC/IRI-PT, prosocial intentions.

Bivariate correlations showed that belief was positively correlated with IRI-EC (*r* = 0.21, *p* <.001) and prosocial intentions (*r* = 0.21, *p* < .001) while negatively correlated with CRT (*r* = -0.24, *p* < .001). There was no significant relationship between belief and the IRI-PT (*r* = 0.02, *p* = *ns*). IRI-EC was negatively correlated with the CRT (*r* = 0.1, *p* < .05)

We entered all variables into a 4-step hierarchical regression analysis predicting belief. Entering demographics into the first step provided a statistically significant model (ΔR^2^ = .028, Δ*F* (3, 523) = 4.942, *p* < .01) and produced a small effect size (Cohens f^2^ = 0.029). Entering IRI-EC and prosocial intentions into the second step significantly improved the model (ΔR^2^ = .046, Δ*F* (2, 521) = 13.028, *p* < .001) and together produced a small effect size (Cohens f^2^ = 0.05). Entering CRT into the third step significantly improved the model (ΔR^2^ = .044, Δ*F* (1, 520) = 25.673, *p* < .001) and produced a small effect size (Cohens f^2^ = 0.049). Entering IRI-PT into the final step significantly improved the model (ΔR^2^ = .012, Δ*F* (1, 519) = 7.313, *p* < .01) but failed to produce a small effect size (Cohens f^2^ = 0.015). The final step of the regression analysis revealed that belief was positively predicted by both empathic concern (*β* = 0.18, *p*< .001) and prosocial intentions (*β* = 0.15, *p*< .001), and negatively predicted by analytic reasoning (*β* = -0.22, *p*< .001) and mentalizing (*β* = -0.13, *p*< .001). No other variables were significant: gender (*β* = 0.00, *p* = .97), age (*β* = 0.07, *p* = .09), education (*β* = 0.02, *p* = .69).

Study 4 extends the results of Studies 1–3 by establishing a positive relationship between belief and prosocial intentions, in addition to empathic concern. Unlike studies 1–3, in study 4 analytic reasoning still predicted disbelief after other variables were taken into account. This may be due to the larger sample used in this study. Finally, and most significantly, findings from Study 4 contradict prior claims about the positive association between mentalizing and belief. Once measures of empathic concern, prosocial intentions and analytic thinking are taken into account, mentalizing (IRI-PT) is found to negatively predict belief.

### Study 5

Study 5 extends Study 4 beyond self-report measures to also include a peer reported measure of empathy [93] and objective (e.g., performance based) measures of mentalizing. A representative item from this peer-report measure is: “understands others by listening attentively”. In addition, we included a self-report measure of the ASD phenotype found to negatively predict belief in prior studies [8]. CRT and IPT were included as measures of analytic thinking. Participants were 69 college students from Case Western Reserve University.

We focus on reporting correlations with belief because of the large number of variables included in this study. Bivariate correlations showed that belief was positively correlated with IRI-EC (*r* = 0.43, *p* <.001) and peer-reports of empathy (*r* = 0.37, *p* <.01) and negatively correlated with the measure of ASD (*r* = -0.40, *p* <.001). All other variables failed to significantly relate to belief. The self and peer report measures of empathy all showed modest negative correlations with the two analytic thinking measures (CRT and IPT), however only one of the four pairwise comparisons was significant (IRI-EC and IPT; (*r* = -0.27, *p* <.05)).

Participants completed these measures over several different sessions in the laboratory.

We conducted a four-step hierarchical multiple regression predicting belief. Entering gender and age into the first step failed to produce a significant model (ΔR^2^ = .05, Δ*F* (2, 66) = 1.751, *p* = .182) but did produce a small effect size (Cohens f^2^ = 0.053). Entering IRI-EC and peer rated empathy [93] into the second step significantly improved the model (ΔR^2^ = .206, Δ*F* (2, 64) = 8.857, *p* < .001) and together these variables produced a medium effect size (Cohens f^2^ = 0.277). Entering the measures of analytic thinking (CRT and IPT) into the third step failed to significantly improve the model (ΔR^2^ = .029, Δ*F* (2, 62) = 1.278, *p* = .278) but together these variables produced a small effect size (Cohens f^2^ = 0.042) The only significant variables at this step were IRI-EC (*β* = .34, *p* < .01) and peer-rated empathy (*β* = .28, *p* < .05),The fourth step included all measures of mentalizing: Imposing Memory Task [94], Social Stories questionnaire [95], Interpersonal Perception Task [96], Reading the Mind in the Eyes [36], DANVA [97] and the Autism Quotient [98]. This step failed to significantly improve the model (ΔR^2^ = .062, Δ*F* (6, 56) = 0.892, *p* = .507) but together these variables produced a small effect size (Cohens f^2^ = 0.095)

IRI-EC was the only variable which remained significant at the final step (*β* = 0.34, *p* < .05). No other individual difference measures added in the third or fourth steps were significant (all *p*’s > 0.16), see S1 Supporting Information.

Similar to studies 3 and 4, Study 5 extended further the link between belief and empathic concern. The best regression model was the second step, where we found that both IRI-EC and peer-empathy independently and significantly predicted belief.

Study 5 found no evidence that measures of mentalizing predict belief once measures of moral concern are taken into account. The Autism Quotient (AQ) used in both this study and in four studies previously reported studies [8], did show a significant bivariate correlation with belief; however, this metric was not significant in the regression analysis after measures which more specifically target empathy were included.

### Study 6

Study 6 extended the assessment of the ASD phenotype using two other measures previously investigated alongside belief [8]: the empathizing quotient (EQ) and the systemizing quotient (SQ) [27]. The CRT and IPT were used as measures of analytic thinking. The Interpersonal Reactivity Index—Perspective Taking subscale (IRI-PT) was used as an additional measure of mentalizing. Participants were a sample of 459 adults from the USA, recruited through AMT.

The systemizing quotient is omitted from subsequent analyses due to low internal consistency (Cronbach’s α = 0.30). This variable was not a significant predictor of belief in a personal God in a prior study employing both the EQ and SQ [8].

Participants completed all measures in the following order: CRT, IRI-PT/IRI-EC, IPT, EQ/SQ, demographics and belief.

Bivariate correlations showed that belief was positively correlated with IRI-EC (*r* = 0.21, *p* <.001) and EQ (*r* = 0.16, *p* <.001). It was negatively correlated with CRT (*r* = -0.16, *p* <.001) and IPT (*r* = -0.15, *p* <.001). CRT was negatively correlated with EQ (*r* = -0.15, *p* <.001)

No other variables reached significance with religious belief, and neither measure of analytic thinking reached significance with IRI-EC.

We entered all variables into a 4-step hierarchical regression analysis predicting belief. Entering demographics into the first step failed to produce a significant model (ΔR^2^ = .01, Δ*F* (3, 455) = 1.472, *p* = .221) or a small effect size (Cohens f^2^ = 0.01). Entering IRI-EC into the second step significantly improved the model (ΔR^2^ = .037, Δ*F* (1, 454) = 17.598, *p* <.001) and produced a small effect size (Cohens f^2^ = 0.039). Entering CRT and IPT into the third step significantly improved the model (ΔR^2^ = .034, Δ*F* (2, 452) = 8.380, *p* <.001) and together these variables produced a small effect size (Cohens f^2^ = 0.037). Adding the EQ in the final step did not significantly improve the model (ΔR^2^ = .009, Δ*F* (2, 450) = 2.142, *p* = .119) and failed to produce a small effect size (Cohens f^2^ = 0.009). The final step of the regression revealed that IRI-EC (*β* = 0.20, *p*< .001) positively predicted belief and both measures of analytic reasoning negatively predicted belief (CRT: *β* = -0.11, *p*< .05; IPT: *β* = -0.11, *p*< .05). No other variables were significant predictors: IRI-PT (*β* = -0.08, *p* = .16), EQ (*β* = 0.09, *p* = .08), gender (*β* = -0.05, *p* = .35), age (*β* = 0.08, *p* = .09), and education (*β* = 0.02, *p* = .62).

Similar to studies 4 & 5, Study 6 provides evidence that once the opposing effects of empathy and analytic thinking are taken into account, mentalizing (as assessed by the EQ and IRI-PT) is no longer a significant predictor of belief. It is noteworthy that the empathizing quotient did not add any predictive power of its own, despite prior published findings [8]. This might be attributed to the nature of the scale, which is comprised of items assessing both mentalizing and empathic concern. As in study 5, once measures which more specifically target empathic concern were included, no evidence of a relationship with the ASD phenotype was found.

### Study 7

Studies 4–6 used direct measures of mentalizing, and found no positive association with religious and spiritual belief. However, prior work has associated loneliness to belief in supernatural agents, a result which is attributed to a general tendency for loneliness to cause increased attributions of agency in a variety of contexts [99, 100]. Study 7 used a 20-item measure of loneliness [101] as an indirect measure of the tendency to attribute agency. We also included two measures of depression (QIDS[102] and DASS-21[103]) because these are linked with loneliness and might account for any observed relationship that loneliness bears to religious and spiritual belief. Participants were a sample of 155 adults from any country other than the USA recruited through AMT.

Participants completed all measures in the following order: Demographics, IRI-EC, belief, loneliness, DASS-21, QIDS.

Bivariate correlations showed that belief was positively related to IRI-EC (*r* = 0.32, *p* <.001) and negatively related to both loneliness (*r* = -0.26, *p* <.001) and depression (*r* = -0.2, *p* <.05).

We entered all variables into a 3-step hierarchical regression analysis predicting belief. Entering demographics significantly improved the model (ΔR^2^ = .06, Δ*F* (3, 151) = 3.218, *p* <.05) and provided a small effect size (Cohens f^2^ = 0.064). Entering IRI-EC into the second step significantly improved the model (ΔR^2^ = .066, Δ*F* (1, 150) = 11.268, *p* <.001) and produced a small effect size (Cohens f^2^ = 0.076). Entering the measure of loneliness and two measures of depression into the final step failed to significantly improve the model (ΔR^2^ = .021, Δ*F* (3, 147) = 1.207, *p* = .309) but together these variables produced a small effect size (Cohens f^2^ = 0.076). The final step revealed that belief was positively predicted by empathic concern (*β* = 0.23, *p*< .01). No other variables were significant: loneliness (*β* = -0.15, *p* = .21), QIDS (*β* = 0.02, *p* = .81), depression (*β* = -0.03, *p* = .20), gender (*β* = 0.06, *p* = .46), age (*β* = 0.08, *p* = .37) and education (*β* = 0.08, *p* = .35).

Study 7 contradicts the view that spiritual and religious belief is predicted by loneliness. Since loneliness has been positively associated with the tendency to perceive agency in other contexts (e.g. animals and gadgets), this finding further undermines the claim that there is a positive association between mentalizing and belief. In contrast, the relationship between belief and empathic concern appears to be highly robust.

### Study 8

The prior seven studies provide compelling evidence that religious belief is positively associated with various measures of empathy and negatively associated with analytic thinking. Study 8 addresses concerns that these findings might be explained by socially desirable responding [104], and/or mediated by social contact associated with religious affiliation. Hence in addition to measures of empathic concern (IRI-EC) and analytic reasoning (CRT), we included measures of socially desirable responding, as well as questions about religious/spiritual practices and attendance at social events.

Questions about belief and attendance were at the very end, after participants had completed all other measures. The item assessing practice was “How often do you attend religious services, pray or meditate with others”, with 6 possible responses ranging from ‘Never’ to “Daily’. The item assessing attendance was “How often do you attend social events organized on behalf of your church or religious group?” The social desirability scale is comprised of two subscales: The Attribution scale, which assesses the degree to which individuals attribute socially desirable behaviors to themselves, and the Denial subscale, which assesses the degree to which they deny socially undesirable behaviors. Representative items for each are as follows, respectively: “I always try to practice what I preach”, and “At times I have really insisted on having things my own way.” Participants were a sample of 371 adults recruited from any country (including the USA) through AMT.

Participants completed all measures in the following order: CRT, IRI -EC, Social Desirability, belief and demographics.

Bivariate correlations showed that belief was positively related to IRI-EC (*r* = 0.3, *p* <.001), socially desirable responses (attribution scale: *r* = 0.26, *p* <.001; denial scale: *r* = 0.19, *p* <.001), attendance/prayers/meditations (*r* = 0.54, *p* <.001) and frequency of attendance at religious affiliated events (*r* = 0.43, *p* <.001). The negative relationship between CRT and IRI-EC was trending towards significance (*r* = -0.1, *p* = .055).

We entered all variables into a 5-step hierarchical regression analysis predicting belief. Entering demographics into the first step failed to produce a significant model (ΔR^2^ = .018, Δ*F* (3, 366) = 2.224, *p* = .085) or a small effect size (Cohens f^2^ = 0.018). Entering IRI-EC into the second step significantly improved this model (ΔR^2^ = .079, Δ*F* (1, 365) = 31.764, *p* <.001) and produced a small effect size (Cohens f^2^ = 0.087). Adding CRT into the next step significantly improved the model (ΔR^2^ = .018, Δ*F* (1, 364) = 7.433, *p* <.01) and produced a small effect size (Cohens f^2^ = 0.02). Entering the two measures of socially desirable responding in the fourth step significantly improved the model (ΔR^2^ = .028, Δ*F* (2, 362) = 5.911, *p* <.01) and produced a small effect size (Cohens f^2^ = 0.032). Adding the two questions assessing attendance and affiliation significantly improved the model (ΔR^2^ = .218, Δ*F* (2, 360) = 61.345, *p* <.001) and produced a medium effect size (Cohens f^2^ = 0.341). The final step of the regression revealed that belief was positively predicted by empathic concern (*β* = 0.16, *p*<.001), frequency of religious and spiritual practice/prayer/meditation (*β* = 0.47, *p* <.001), and age (*β* = 0.09, *p*<.05); and negatively predicted by gender (*β* = -0.10, *p* <.05) and education (*β* = -0.09, *p* < .05). No other variables were significant predictors: CRT (*β* = -0.05, *p* = .30), Attribution (*β* = -0.02, *p* = .77), Denial (*β* = 0.00, *p* = .96) and frequency of attendance at religious affiliated events (*β* = 0.08, *p* = .23).

At the suggestion of an anonymous reviewer, we re-ran the regression analysis predicting empathic concern (IRI-EC), as this could help clarify whether empathy is better predicted by religious belief or by religious practice. The final step of this regression showed that empathy was independently and significantly predicted by belief (*β* = 0.20, *p* <.001), Attribution (*β* = 0.20, *p* <.01), Denial (*β* = 0.12, *p* < .05) and gender (*β* = -0.18), *p* < .001). No other variables were significant: Age (*β* = 0.07, *p* = .15), education (*β* = -0.05, *p* = .31), CRT (*β* = 0.00, *p* = .96), attendance/prayer/meditation (*β* = 0.00, *p* = .96), social events linked to religious affiliation (*β* = 0.01, *p* = .93).

These data serve to both replicate and extend the above studies by demonstrating that neither socially desirable responding, religious practice, nor social contact associated with religious affiliation explains the positive relationship between moral concern and belief.

### Pooled Analysis

Prior work demonstrates a small but consistent gender gap in religious belief, with females tending to hold more to religious and spiritual worldviews [105]. A prior study, using some of the same measures used here, has reported that this gender difference can be explained by gender differences in mentalizing [8]. However, prior work has shown a that males have a stronger tendency towards mentalizing, and females towards empathic concern [26, 106]. The current findings thus raise the possibility that the gender gap in religious belief may be best explained by gender differences in empathic concern rather than gender differences in mentalizing.

We conducted a pooled analysis of data from Studies 1, 2, 3, 4, 6 & 8 (N = 1984) to address two questions:

1. Given that females tend to both have higher levels of empathic concern and tend to be more religious than males, can the gender gap in religious belief be accounted for by gender differences in moral concern?
2. Given inconsistent findings concerning the relationship between analytic thinking and belief, what are the relative magnitudes of the associations with empathic concern and analytic thinking?

To assess these questions, we conducted a 3-step hierarchical linear regression using those variables common between the studies to predict religious and spiritual belief.

In the first step, we entered just demographic variables, and added dummy variables to account for any differences between the samples/studies. This produced a significant model (ΔR^2^ = .032, Δ*F* (8, 1975) = 8.074, *p* <.001) and together the variables had a small effect size (Cohens f^2^ = 0.033). We found that both female gender (*β* = 0.06, *p* <.05) and age (*β* = 0.09, *p* <.001) were positive predictors. Education (*β* = -0.01, *p* = 0.70) was not significant.

In the second step, we added empathic concern (IRI-EC), which positively predicted belief (*β* = 0.25, *p* <.001). This significantly improved the model (ΔR^2^ = .058, Δ*F* (1, 1974) = 125.134, *p* <.001) and produced a small effect size (Cohens f^2^ = 0.063). Providing an answer to our first question, gender no longer significantly predicted belief (*β* = 0.01, *p* = 0.57), while age remained significant (*β* = 0.06, *p* <.05) and education remained insignificant (*β* = 0.01, *p* = 0.79).

In the third step we added analytic thinking (CRT), which negatively predicted belief (*β* = -0.18, *p* <0.001). This significantly improved the model (ΔR^2^ = .029, Δ*F* (1, 1973) = 65.753, *p* <.001) and produced a small effect size (Cohens f^2^ = 0.035). In this final step, empathic concern continued to demonstrate a stronger positive effect on belief (*β* = 0.23, *p* <0.001), as did age (*β* = 0.06, *p* <0.005). Education (*β* = 0.03, *p* = 0.19) and Gender (*β* = -0.01, *p* = 0.63) remained insignificant.

To address our second question, we examined both bivariate and partial correlations between belief, analytic thinking, and empathic concern. Bivariate correlations between the variables were as follows: Belief and CRT (*r* = -0.19, *p* <0.001); Belief and IRI-EC, (*r* = 0.26, *p* <0.001); CRT and IRI-EC (*r* = -0.11, *p* <0.001). The correlation between belief and IRI-EC was found to be stronger than the correlation between belief and CRT using Hotelling’s *t*-test (*t* = 14.1, *df* = 1981, *p* <0.001). Partial correlations (controlling gender, age and education) were as follows: Belief and CRT (*r* = -0.19, *p* <0.001); Belief and IRI-EC, (*r* = 0.24, *p* <0.001); CRT and IRI-EC (*r* = -0.09, *p* <0.001. The correlation between belief and IRI-EC was found to be stronger than the correlation between belief and CRT using Hotelling’s *t*-test (*t* = 13.5, *df* = 1981, *p* <0.001). These findings indicate that IRI-EC explains more variance in religious and spiritual belief than the CRT, with the amount of additional variance explained estimated at 87% for the bivariate comparison and 60% for the partial correlation comparison.

## General Discussion

Religious and spiritual belief have been positively associated with social and emotional cognition [8–22] and negatively associated with measures of analytic thinking [1, 4–8]. The present studies make two key contributions to the current literature. First, we distinguish between different dimensions of social cognition, and second we assess their association with belief while also controlling for their negative association with analytic thinking. According to our theoretical model, moral concern represents one broad dimension of social cognition distinct from mentalizing [24, 29, 30, 46]. This view is supported by the observation that moral concern and mentalizing relate to distinct neuropsychological profiles, such that a deficit in moral concern is the primary personality characteristic of psychopathy [33, 37, 38], whereas a deficit in mentalizing is thought to be a key characteristic of Autism Spectrum Disorders [32, 33, 35]. To our knowledge, this is the first series of studies to simultaneously test these three cognitive constructs—analytic thinking, moral concern, and mentalizing—in order to test the extent to which each independently contributes to religious and spiritual belief.

The studies presented here establish a clear positive association between moral concern, using a variety of specific measures which represent components of this broader construct, and belief in God and/or a universal spirit. This relationship was found to be robust even when controlling for the previously established link between analytic thinking and religious disbelief. Further, no evidence was found supporting the view that either direct or indirect measures of mentalizing predict belief after taking into account measures of moral concern.

We report eight studies (total n = 2212), including seven online studies and one laboratory based study. In every study we found that a central aspect of moral concern, empathic concern (IRI-EC), significantly predicted religious and spiritual belief. This relationship was found both for bivariate correlations and after entering all other variables into a regression analysis. In three of the studies (3, 4 & 5), we found that belief was additionally positively predicted by a second measure of moral concern (IWAH_Global, prosocial intentions, peer-reported empathy). In our final study, we demonstrated that the link between empathic concern and belief remained after controlling for socially desirable responding, religious attendance/practice and social affiliation with religion.

Of the seven studies which included measures of analytic thinking, all demonstrated a negative relationship with belief in the bivariate correlations; however, this relationship only remained significant in 2 out of the 7 studies after entering other variables into the regression analyses. We conducted a pooled analysis to establish whether or not a measure of analytic thinking (CRT) remained significant after accounting for its negative relationship with empathic concern (IRI-EC). We found that CRT performance did account for variance in belief; however, the effect was significantly smaller than the positive relationship observed with the IRI-EC.

Four of the eight studies included measures of mentalizing. We used 3 direct self-report measures of mentalizing, 5 direct objective (or performance based) measures of mentalizing, and one indirect self-report measure (loneliness). None of the four studies demonstrated a positive relationship between belief and any of the nine measures of mentalizing after other variables were taken into account in the regression analysis. These findings suggest that any positive relationship with mentalizing is considerably weaker than the positive relationship with moral concern. Further, it appears that some measures of mentalizing associated with the Autistic phenotype (AQ and EQ) may have previously been interpreted as demonstrating an association with belief because they fail to fully distinguish mentalizing from moral concern. This is suggested by our findings in Studies 5 and 6 in which AQ and EQ demonstrated relationships with belief in the bivariate correlations but did not survive in the regression analyses after measures of moral concern were taken into account. Hence, the current results suggest that non-believers have personality profiles more closely associated with the psychopathic phenotype (i.e., deficits in moral concern) than with the ASD phenotype (i.e., deficits in mentalizing). Consistent with this view, a large community survey [107] of correlates of psychopathic personality traits concluded that “religious non-believers reported higher levels of psychopathic traits, namely Self-Centered Impulsivity and Coldheartedness, than do religious believers, although only the magnitude for Coldheartedness reached Cohen's cut-off for a small effect size." Similarly, a survey of 312 college students [108]examining the relationship between Religious/Spiritual Well-Being (RSWB) and ‘dark triad’ personality traits found that “RSWB was confirmed to be negatively correlated with these negative aspects of personality, in particular with subclinical psychopathy.”

The relationships observed between belief, analytic thinking, mentalizing and moral concern fit well with a model we have previously presented and supported with neuroscientific evidence [24, 29, 31, 109, 110]. According to that model, analytic thinking and moral concern represent two cognitive modes which our neural architecture causes to be in competition with each other. Analytic thinking is associated with a naturalistic/materialistic worldview, whereas moral concern is associated with a spiritual worldview.

### Limitations

While the present studies have provided good support for hypotheses generated by our model, they are limited in a number of ways.

First, since we rely exclusively on correlational observations, further work is needed to better establish causal relationships between belief, analytic thinking and moral concern, especially when investigated simultaneously. It has been shown that priming analytic thinking reduces religious belief [4, 5]. In addition, it has been shown that priming religious and spiritual thinking increases prosocial behavior [111]. However, we are unaware of any studies which have manipulated both, or which have included measures to assess whether the observed effects are mediated by the hypothesized competitive relationship between analytic thinking and moral concern.

Second, while we employed performance based measures of mentalizing, the measures of moral concern were all subjective (either self or peer-report). More objective measures of moral concern are wanted to further establish their relationship to religious and spiritual belief.

Third, although we assess the impact of analytic thinking and moral concern while controlling for measures of the other variable, it is unlikely that we were able to capture all the variance in either analytic thinking or moral concern using our control variables. In three studies (3, 4, & 5), we found more than one measure of moral concern predicted belief, and in one study (6) we found that more than one measure of analytic thinking predicted disbelief. Hence, for example, it remains possible that the observed relationship between analytic thinking and belief might disappear after fully controlling for moral concern by using a variety of measures. Again, better and more comprehensive measures of moral concern are wanted.

Fourth, the distinct dimensions of social cognition remain to be fully explored. There is evidence that religious thinking and/or belief is linked to emotional intelligence [20, 21] and to emotional self-control [18, 19]. The present findings strongly indicate that measures of moral concern, broadly construed, are better predictors of belief than measures of mentalizing, broadly construed. However, it is not safe to conclude that moral concern is the only or best predictor of religious belief. Further work is needed to subdivide social cognition and establish relationships between dimensions; and also to examine the relationships between distinct dimensions of social cognition and distinct dimensions of non-social reasoning (including but not limited to analytic thinking).

Fifth, researchers have been turning to Amazon Mechanical Turk more and more in recent years, increasing the likelihood that many ‘workers’ complete the same measures in different studies [112]. There are worries that this compromises the validity of the measures, especially the CRT. We did not ask the participants whether they had seen these measures before. Hence, it is possible that familiarity with some measures may have biased results. In this respect, it is worth noting that these studies were conducted between 2011 and 2013, when researchers were just beginning to use the AMT. More generally, researchers investigating the relationship between laboratory findings and those acquired by AMT have shown that, although there are differences, the similarities between the two support the view that AMT provides valid and reliable data, especially when using ‘catch’ questions [112–115]. Finally, the validity of our findings are further supported by the correspondence between our AMT studies and our in lab study (Study 5). The results reported here are also highly consistent with overlapping findings reported by other researchers employing both AMT and laboratory methods [4–6].

### Inconsistencies with Prior Work

A prior study found that belief in a personal god was negatively related to measures of the ASD phenotype and that this relationship was mediated by deficits in mentalizing [8]. In that study, mentalizing was assessed by the empathy quotient (EQ) and reading the mind in the eyes task, both of which were used in the studies reported here. While we did find a positive relationship between belief and EQ in Study 6, the relationship was no longer significant in the regression analysis after taking into account analytic thinking and moral concern. In Study 5, we did not replicate the previously observed relationship [8] between belief and Reading the Mind in the Eyes. It is possible that we did not have the power to detect this relationship. Nevertheless, our data provide evidence that moral concern predicts belief more strongly than previously used measures of mentalizing. A second possible explanation for these inconsistencies is that we assessed belief in “God or a universal spirit”, whereas prior work [8] assessed belief in a personal god. It is thus possible that the nature of religious belief (e.g. the degree to which it is personal) is more influenced by mentalizing than overall level of religious belief. In support of this, other work suggests that the specific form of religious belief (e.g., belief in a benevolent or authoritarian god) alters the relationship with moral concern [116]. Here, we focused on associations between moral concern and religious and spiritual belief more broadly.

A second inconsistency concerns the results of Study 7, where we investigated links between loneliness and belief. Prior work has provided evidence that loneliness increases the perception of agency in inanimate objects and belief in supernatural agents [99]. Correlational evidence showed that higher levels of loneliness increased the tendency to anthropomorphize inanimate gadgets. Causal evidence showed that inducing loneliness by telling participants “You’re the type [of person] who will end up alone later in life” increased belief in supernatural agents. What might explain the inconsistencies between these prior findings and the present work?

First, it is worth noting that the previously reported correlational evidence only provides support for a relationship between loneliness and anthropomorphizing inanimate objects (correlations between loneliness and religious belief were not tested). Second, it is likely that their loneliness induction, which was used to investigate religious belief, had non-specific effects. The induction may have not only encouraged feelings of loneliness, but also feelings of moral concern. It would be beneficial for future work to control for moral concern when conducting experimental manipulations designed to investigate the cognitive origins of religious and spiritual beliefs.

### Broader Significance

These results reported here present a challenge to a number of theoretical accounts of religious belief, especially those which emphasize a link between religious and spiritual beliefs and the perception of agency [8, 11, 16, 17, 22]. The present findings put religious and spiritual beliefs in a new light by suggesting that they are not so much linked to the perception of agency as they are broadly to moral concern, and in particular empathic concern. In line with this view, a number of theologians and religious scholars have claimed that compassion is a central theme that unites many religions [117, 118]. While further work is needed to establish causal links, it is plausible both that religious thinking increases moral concern, and that individuals who possess greater levels of moral concern are more inclined to identify with religious and spiritual worldviews.

## Supporting Information

S1 Data (XLSX)

S1 Supporting Information (DOCX)
