# Correction: Why Do You Believe in God? Relationships between Religious Belief, Analytic Thinking, Mentalizing and Moral Concern

**DOI:** 10.1371/journal.pone.0155283

**Published:** 2016-05-11

**Authors:** Anthony Ian Jack, Jared Parker Friedman, Richard Eleftherios Boyatzis, Scott Nolan Taylor

There was an error in the coding of male and female participants in two studies, and the involvement of these data points in the pooled analysis.

In the Results and Discussion, Study 5 and Study 8 contain the incorrect signs for effects reported on gender.

Additionally, in the “Pooled Analysis” section of the Results and Discussion, there are some changes to the statistics reported in the regression analysis, of the order of <1% in the values.

These differences do not affect the significance of any reported findings or any of the conclusions drawn in the manuscript.

The correlation coefficients remain unchanged, as we clarified in the caption to the tables that 1 = Females and 2 = Males.

## Supporting Information

S1 File (DOCX)
