# Supplementary material for: Why Do You Believe in God? Relationships between Religious Belief, Analytic Thinking, Mentalizing and Moral Concern
Source: PLoS One. 2016 Mar 23;11(3):e0149989. doi: 10.1371/journal.pone.0149989 (PMC4805169; doi:10.1371/journal.pone.0149989)
Supplement: S1 Supporting Information — (DOCX) [file pone.0149989.s002.docx]

**Study 1**

Participants

Three hundred and ninety-five participants were recruited through Amazon’s Mechanical Turk, where they were linked to a survey hosted by Survey Monkey. Participants were required to have completed 50 HITs on Mechanical Turk with an approval rating of 95% or higher. Location of participants was limited to the USA. One hundred and fifty-nine people were excluded from the final analyses for failing to answer our catch-questions correctly, leaving a total of 236 participants with complete data (140 females, 59.3% females; average age 35.05, SD = 13.05). These participants were paid $0.45 for their HIT.

Procedure and measures

Participants first provided demographic information (sex, age, and level of education). Religious and spiritual belief was assessed with a single item measure on a 7-point Likert scale (1= *not at all*; 7 = *definitely yes*): Do you believe in the existence of either God or a universal spirit? Participants then completed the 20-question intuitive physics test (IPT), a multiple-choice test that assesses physical reasoning and the understanding of causal relationships between inanimate objects [1]

Participants then completed the 7-item empathic concern subscale of the Interpersonal Reactivity Index [2]. Items included questions such as “I often have tender, concerned feelings for people less fortunate than me” and “I would describe myself as a pretty softhearted person,” answered on a 5-point Likert scale (1 = *does not describe me well*; 5 = *describes me very well*). This scale had good internal consistency: Cronbach’s α = 0.87.

Finally, participants were presented with the cognitive reflection test (CRT; [3].

Whereas the IPT evaluates implicit knowledge of causal relationships between physical objects – folk-physics – while the CRT evaluates one’s ability to override an intuitively attractive – albeit incorrect – response by engaging in deliberate and reflective reasoning. We computed averages for the final scores for both the IPT and CRT. The survey included additional measures which are not of relevance here.

Demographic variables

Sex, (Males = 1, Females = 2), age in years (range: 18–83), and level of education [1 = some high school or less(2.1%), 2 = high school diploma (11.9%), 3 = some college (28.4%), 4 = associates/2-year degree (8.5%), 5 = bachelor’s/4-year degree (30.9%), 6 = some graduate school (6.4%), 7 = master’s degree (11.0%), 8 = doctorate degree (0.8%)] were also assessed and used as control variables. These distributions were relatively the same for all samples in these studies.

Results

**Table 1.** Means (M), standard deviations (SD), and bivariate correlations among all study variables. N = 236. Gender, 1 = *male*, 2 = *female.* Religious/spiritual belief (belief), empathic concern (IRI-EC) Cognitive Reflection test (CRT), Intuitive Physics Test (IPT).

| **Variable** | **Mean** | **S.D.** | **1** | **2** | **3** | **4** | **5** | **6** |
| --- | --- | --- | --- | --- | --- | --- | --- | --- |
| 1. Gender | 1.59 | 0.49 |  |  |  |  |  |  |
| 2. Age | 35.05 | 13.05 | .141* |  |  |  |  |  |
| 3. Edu | 4.22 | 1.61 | 0.02 | .296** |  |  |  |  |
| 4. Belief | 4.98 | 2.35 | 0.12 | .185** | -0.02 |  |  |  |
| 5. IRI-EC | 3.78 | 0.79 | .288** | .179** | -0.04 | .302** |  |  |
| 6. CRT | 0.46 | 0.41 | -0.10 | 0.06 | .202** | -.146* | -.169** |  |
| 7. IPT | 0.55 | 0.15 | -.197** | 0.05 | .143* | -.134* | -.181** | .310** |
| * Correlation is significant at the 0.05 level (2-tailed). | | | | |  |  |  |  |
| ** Correlation is significant at the 0.01 level (2-tailed). | | | | | |  |  |  |

**Table 2**

Hierarchical linear regression analysis predicting belief with gender, age, education level, empathic concern (IRI-EC), mechanistic reasoning (IPT), and analytic reasoning (CRT). N = 236.

| **Model** |  | **B** | ***S.E.*** | **β** | ***t*** | ***p*** | **R2Δ** | ***FΔ*** | **F sig** | ***Cohens f2*** |
| --- | --- | --- | --- | --- | --- | --- | --- | --- | --- | --- |
| 1 | Intercept | 3.489 | 0.681 |  | 5.126 | 0.000 |  |  |  |  |
|  | Gener | 0.455 | 0.309 | 0.095 | 1.471 | 0.143 |  |  |  |  |
|  | Age | 0.035 | 0.012 | 0.194 | 2.865 | 0.005 |  |  |  |  |
|  | Edu | -0.109 | 0.098 | -0.075 | -1.118 | 0.265 |  |  |  |  |
|  |  |  |  |  |  |  | 0.049 | 3.955 | 0.009 | 0.052 |
| 2 | Intercept | 1.177 | 0.872 |  | 1.349 | 0.179 |  |  |  |  |
|  | Gender | 0.116 | 0.311 | 0.024 | 0.373 | 0.71 |  |  |  |  |
|  | Age | 0.027 | 0.012 | 0.149 | 2.244 | 0.026 |  |  |  |  |
|  | Edu | -0.074 | 0.095 | -0.051 | -0.774 | 0.44 |  |  |  |  |
|  | IRI-EC | 0.789 | 0.195 | 0.266 | 4.047 | 0.000 |  |  |  |  |
| 3 |  |  |  |  |  |  | 0.063 | 16.381 | 0.000 | 0.071 |
|  | Intercept | 2.166 | 1.102 |  | 1.964 | 0.051 |  |  |  |  |
|  | Gender | 0.034 | 0.314 | 0.007 | 0.108 | 0.914 |  |  |  |  |
|  | Age | 0.028 | 0.012 | 0.157 | 2.36 | 0.019 |  |  |  |  |
|  | Edu | -0.038 | 0.097 | -0.026 | -0.395 | 0.693 |  |  |  |  |
|  | IRI-EC | 0.723 | 0.197 | 0.244 | 3.665 | 0.000 |  |  |  |  |
|  | CRT | -0.503 | 0.382 | -0.087 | -1.317 | 0.189 |  |  |  |  |
|  | IPT | -1.055 | 1.077 | -0.065 | -0.98 | 0.328 |  |  |  |  |
|  |  |  |  |  |  |  | 0.014 | 1.804 | 0.167 | 0.015 |

**Study 2**

Participants

Two hundred and eighty-two participants were recruited through Amazon’s Mechanical Turk, where they were linked to a survey hosted by SurveyMonkey. Participants were required to have completed 50 HITs on Mechanical Turk with an approval rating of 95% or higher. Location of participants was limited to the USA. Forty-nine people were excluded from the final analyses for failing to answer our catch-questions correctly, leaving a total of 233 participants with complete data (131 females, 56.2% females; average age 35.19, SD = 12.10). These participants were paid $0.85 for their HIT.

Procedure and measures

Study 2 was identical to Study 1 except for the addition of 16-items from the Mill-Hill Vocabulary Scale [4] to measure crystalized intelligence. Participants were presented a bolded-word with five words underneath, and prompted to “select one of the five definitions that best fits the word in bold.” The CI measure was placed after the IRI-EC and before the CRT. Final scores for the vocabulary test were averaged, just as they were for the IPT and CRT.

The measure of empathic concern was found to have good internal consistency (Cronbach’s α = 0.88). The survey included additional measures which are not of relevance here.

Demographic variables

Sex, (Males = 1, Females = 2), age in years (range: 18-74), and level of education [1 = some high school or less (1.7%), 2 = high school diploma (9.9%), 3 = some college (31.3%), 4 = associates/2-year degree (9.9%), 5 = bachelor’s/4-year degree (33.5%), 6 = some graduate school (5.2%), 7 = master’s degree (7.3%), 8 = doctorate degree (1.3%)] were also assessed and used as control variables.

Results

**Table 3**. Means (M), standard deviations (SD), and bivariate correlations among study variables. N = 233. Gender, 1 = *male*, 2 = *female.* Empathic concern (IRI-EC), mechanistic reasoning (IPT), analytic reasoning (CRT) and crystallized intelligence (CI).

| **Variable** | **Mean** | **S.D.** | **1** | **2** | **3** | **4** | **5** | **6** | **7** |
| --- | --- | --- | --- | --- | --- | --- | --- | --- | --- |
| 1. Gender | 1.56 | 0.50 |  |  |  |  |  |  |  |
| 2. Age | 35.19 | 12.10 | 0.032 |  |  |  |  |  |  |
| 3. Edu | 4.15 | 1.50 | 0.103 | .182** |  |  |  |  |  |
| 4. Belief | 4.98 | 2.40 | .148* | 0.091 | -0.049 |  |  |  |  |
| 5. IRI-EC | 3.82 | 0.80 | .264** | 0.118 | -0.046 | .352** |  |  |  |
| 6. CRT | 0.40 | 0.41 | -.146* | -0.013 | .163* | -.245** | -.220** |  |  |
| 7. IPT | 0.54 | 0.15 | -.272** | 0.088 | 0.018 | -.161* | -.230** | .363** |  |
| 8. CI | 0.49 | 0.25 | 0.08 | .245** | .236** | -.189** | 0.043 | .268** | .248** |
| * Correlation is significant at the 0.05 level (2-tailed). | | | | |  |  |  |  |  |
| ** Correlation is significant at the 0.01 level (2-tailed). | | | | | |  |  |  |  |

**Table 4**

Linear regression analysis predicting belief with gender, age, education, empathic concern (IRI-EC), mechanistic reasoning (IPT), analytic reasoning (CRT) and crystallized intelligence (CI). N = 233.

| **Model** |  | **B** | ***S.E.*** | **β** | ***t*** | ***p*** | **R2Δ** | ***FΔ*** | **F sig** | ***Cohens f2*** |
| --- | --- | --- | --- | --- | --- | --- | --- | --- | --- | --- |
| 1 | Intercept | 3.677 | 0.74 |  | 4.966 | 0.000 |  |  |  |  |
|  | Gender | 0.738 | 0.315 | 0.153 | 2.345 | 0.02 |  |  |  |  |
|  | Age | 0.02 | 0.013 | 0.101 | 1.527 | 0.128 |  |  |  |  |
|  | education | -0.133 | 0.106 | -0.084 | -1.261 | 0.209 |  |  |  |  |
|  |  |  |  |  |  |  | 0.036 | 2.837 | 0.039 | 0.037 |
| 2 | Intercept | 0.704 | 0.919 |  | 0.766 | 0.444 |  |  |  |  |
|  | Gender | 0.313 | 0.311 | 0.065 | 1.008 | 0.315 |  |  |  |  |
|  | Age | 0.012 | 0.013 | 0.059 | 0.94 | 0.348 |  |  |  |  |
|  | education | -0.083 | 0.101 | -0.052 | -0.821 | 0.412 |  |  |  |  |
|  | IRI-EC | 0.972 | 0.193 | 0.326 | 5.038 | 0.000 |  |  |  |  |
|  |  |  |  |  |  |  | 0.097 | 25.379 | 0.000 | 0.111 |
| 3 | Intercept | 1.36 | 1.154 |  | 1.178 | 0.24 |  |  |  |  |
|  | Gender | 0.311 | 0.312 | 0.064 | 0.995 | 0.321 |  |  |  |  |
|  | Age | 0.019 | 0.013 | 0.098 | 1.553 | 0.122 |  |  |  |  |
|  | education | 0.012 | 0.101 | 0.008 | 0.12 | 0.905 |  |  |  |  |
|  | IRI-EC | 0.925 | 0.193 | 0.31 | 4.799 | 0.000 |  |  |  |  |
|  | CRT | -0.693 | 0.399 | -0.117 | -1.737 | 0.084 |  |  |  |  |
|  | IPT | 0.195 | 1.072 | 0.013 | 0.182 | 0.856 |  |  |  |  |
|  | Vocab (CI) | -0.124 | 0.04 | -0.205 | -3.062 | 0.002 |  |  |  |  |
|  |  |  |  |  |  |  | 0.061 | 5.631 | 0.001 | 0.076 |

**Study 3**

Participants

Two hundred and seventy-seven participants were recruited through Amazon’s Mechanical Turk, where they were linked to a survey hosted by SurveyMonkey. Participants were required to have completed 500 HITs on Mechanical Turk with an approval rating of 97% or higher. Participation was restricted to the United States. One hundred and eighteen people were excluded from the final analyses for failing to answer our catch-questions correctly, leaving a total of 159 participants with complete data (55 females, 34.6% females; average age 32.13, SD = 9.74). Participants were paid $0.45 for their HIT.

Procedure and measures

Participants first completed the self-report measure of empathic concern (IRI-EC), which had acceptable internal consistency (Cronbach’s α = .72), followed by the CRT. Next, participants completed the Identification With All Humanity scale (IWAH; [5]. The IWAH is a 9-item self-report measure assessing individual differences in the extent to which one feels moral concern for out-group members. Each question asks the participant to indicate with a 5-point scale (1 = Not at all, 2 = Just a little, 3 = Somewhat, 4 = Quite a bit, 5 = Very much) how much they identify with “people in my community”, “people in my country”, and “All of humanity”. A representative item is: “How much do you identify with (that is, feel a part of, feel love toward, have concern for) each of the following.” We only report scores for the “all of humanity subscale”, as this is the most relevant to the theoretical predictions under investigation.

Participants then answered two questions assessing their political stance (liberal or conservative) on social and economic issues [6]. Responses were scored with a 7-point Likert scale (1= Very liberal, 7 = Very conservative). Finally, participants completed the same single item measure of belief, followed by demographic questions.

Demographic variables

Sex, (Males = 1, Females = 2), age in years (range: 19-68), and level of education [1 = some high school or less (1.3%), 2 = high school diploma (2.5%), 3 = some college (12.6%), 4 = associates/2-year degree (1.3%), 5 = bachelor’s/4-year degree (44.0%), 6 = some graduate school (3.1%), 7 = master’s degree (34.6%), 8 = doctorate degree (0.6%), 0 = other (0.0%)] were also assessed and used as control variables.

Results and discussion

**Table 5.** Mean (M), standard deviations (SD), and bivariate correlations among study variables. N=159. Gender, 1 = *male*, 2 = *female*. Empathic concern (IRI-EC), universal moral concern (IWAH_Global), analytic reasoning (CRT), and two items about social issues and economic issues

| **Variable** | **Mean** | **S.D.** | **1** | **2** | **3** | **4** | **5** | **6** | **7** | **8** |
| --- | --- | --- | --- | --- | --- | --- | --- | --- | --- | --- |
| 1. Gender | 1.35 | 0.48 |  |  |  |  |  |  |  |  |
| 2. Age | 32.13 | 9.74 | 0.051 |  |  |  |  |  |  |  |
| 3. Edu | 5.35 | 1.52 | 0.067 | 0.078 |  |  |  |  |  |  |
| 4. Belief | 5.82 | 1.70 | 0.031 | -0.028 | 0.082 |  |  |  |  |  |
| 5. IRI-EC | 3.83 | 0.60 | 0.091 | 0.081 | -0.099 | .276** |  |  |  |  |
| 6. IWAH_Global | 3.71 | 0.76 | -0.067 | 0.012 | 0.02 | .339** | .303** |  |  |  |
| 7. CRT | 0.56 | 0.42 | -0.057 | -0.056 | 0.02 | -.215** | 0.016 | -.237** |  |  |
| 8. Social_Issues | 3.66 | 1.60 | -0.085 | -0.083 | 0.003 | 0.147 | -.203* | 0.061 | -.278** |  |
| 9. Economic Issues | 3.96 | 1.47 | -0.108 | -0.089 | -0.042 | 0.012 | -0.135 | 0.091 | -.267** | .658** |
| ** Correlation is significant at the 0.01 level (2-tailed). | | | | | |  |  |  |  |  |
| * Correlation is significant at the 0.05 level (2-tailed). | | | | |  |  |  |  |  |  |

**Table 6.** Linear regression analysis predicting belief with gender, age, and education, empathic concern (IRI-EC), universal moral concern (IWAH_Global), analytic reasoning (CRT), and two items about social issues and economic issues. N = 159.

| **Model** |  | **B** | ***S.E.*** | **β** | ***t*** | ***p*** | **R2Δ** | ***FΔ*** | **F sig** | ***Cohens f2*** |
| --- | --- | --- | --- | --- | --- | --- | --- | --- | --- | --- |
| 1 | Intercept | 5.392 | 0.727 |  | 7.416 | 0.000 |  |  |  |  |
|  | Gender | 0.099 | 0.286 | 0.028 | 0.347 | 0.729 |  |  |  |  |
|  | Age | -0.006 | 0.014 | -0.036 | -0.447 | 0.655 |  |  |  |  |
|  | Edu | 0.092 | 0.09 | 0.082 | 1.026 | 0.307 |  |  |  |  |
|  |  |  |  |  |  |  | 0.009 | 0.448 | 0.719 | 0.009 |
| 2 | Intercept | 0.893 | 1.113 |  | 0.803 | 0.423 |  |  |  |  |
|  | Gender | 0.1 | 0.267 | 0.028 | 0.374 | 0.709 |  |  |  |  |
|  | Age | -0.01 | 0.013 | -0.057 | -0.765 | 0.446 |  |  |  |  |
|  | Edu | 0.11 | 0.084 | 0.099 | 1.314 | 0.191 |  |  |  |  |
|  | IRI-EC | 0.575 | 0.223 | 0.204 | 2.576 | 0.011 |  |  |  |  |
|  | IWAH_global | 0.625 | 0.176 | 0.278 | 3.553 | 0.001 |  |  |  |  |
|  |  |  |  |  |  |  | 0.152 | 13.894 | 0.000 | 0.181 |
| 3 | Intercept | 1.535 | 1.137 |  | 1.35 | 0.179 |  |  |  |  |
|  | Gender | 0.048 | 0.265 | 0.013 | 0.181 | 0.856 |  |  |  |  |
|  | Age | -0.012 | 0.013 | -0.068 | -0.914 | 0.362 |  |  |  |  |
|  | Edu | 0.119 | 0.083 | 0.107 | 1.438 | 0.153 |  |  |  |  |
|  | IRI-EC | 0.631 | 0.222 | 0.224 | 2.844 | 0.005 |  |  |  |  |
|  | IWAH_global | 0.519 | 0.18 | 0.231 | 2.881 | 0.005 |  |  |  |  |
|  | CRT | -0.685 | 0.309 | -0.169 | -2.216 | 0.028 |  |  |  |  |
|  |  |  |  |  |  |  | 0.026 | 4.91 | 0.028 | 0.032 |
| 4 | Intercept | 1.048 | 1.275 |  | 0.823 | 0.412 |  |  |  |  |
|  | Gender | 0.053 | 0.262 | 0.015 | 0.202 | 0.841 |  |  |  |  |
|  | Age | -0.011 | 0.013 | -0.063 | -0.862 | 0.39 |  |  |  |  |
|  | Edu | 0.112 | 0.082 | 0.1 | 1.369 | 0.173 |  |  |  |  |
|  | IRI-EC | 0.707 | 0.223 | 0.251 | 3.177 | 0.002 |  |  |  |  |
|  | IWAH_global | 0.515 | 0.177 | 0.229 | 2.902 | 0.004 |  |  |  |  |
|  | CRT | -0.593 | 0.318 | -0.146 | -1.866 | 0.064 |  |  |  |  |
|  | socialissues | 0.276 | 0.104 | 0.26 | 2.664 | 0.009 |  |  |  |  |
|  | economicissues | -0.213 | 0.112 | -0.185 | -1.909 | 0.058 |  |  |  |  |
|  |  |  |  |  |  |  | 0.037 | 3.608 | 0.029 | 0.049 |

**Study 4**

Participants

Seven hundred and five participants were recruited through Amazon’s Mechanical Turk, where they were linked to a survey hosted by SurveyMonkey. Participants were required to have completed 500 HITs on Mechanical Turk with an approval rating of 95% or higher. There was no geographical restriction for participation. One hundred and seventy-eight people were excluded from the final analyses for failing to answer our catch-questions correctly, leaving a total of 527 participants with complete data (275 females, 52.2% females; average age 30.29, SD = 10.17). Participants were paid $0.44 for their HIT.

Procedures and measures

Participants were first asked demographic questions (age, gender, education) and the single-item measure of belief. They then completed the Cognitive Reflection Test, followed by the empathic concern (IRI-EC) and perspective taking (IRI-PT) subscales of the Interpersonal Reactivity Index [2]. Both of these scales had good internal consistency: IRI-EC, Cronbach’s α = 0.88; IRI-PT, Cronbach’s α = 0.81. Participants then completed a six-item measure assessing prosocial intentions [7], which had good internal consistency Cronbach’s α = 0.88. The survey included additional measures which are not of relevance here.

Demographic variables

Sex, (Males = 1, Females = 2), age in years (range: 19-67), and level of education [1 = some high school or less (0.4%), 2 = high school diploma (8.7%), 3 = some college (30.6%), 4 = associates/2-year degree (8.2%), 5 = bachelor’s/4-year degree (30.9%), 6 = some graduate school (4.0%), 7 = master’s degree (14.8%), 8 = doctorate degree (2.5%), 0 = other (0.0%)] were also assessed and used as control variables.

Results

**Table 7.** Mean (M), standard deviations (SD), and bivariate correlations among study variables. N=527. Gender, 1 = *male*, 2 = *female*. Empathic concern (IRI-EC), prosocial intentions (Pro_Int), analytic reasoning (CRT), and perspective taking (IRI-PT).

| **Variable** | **Mean** | **S.D.** | **1** | **2** | **3** | **4** | **5** | **6** | **7** |
| --- | --- | --- | --- | --- | --- | --- | --- | --- | --- |
| 1. Belief | 4.61 | 2.47 |  |  |  |  |  |  |  |
| 2. Gender | 1.52 | 0.50 | .127** |  |  |  |  |  |  |
| 3. Age | 30.29 | 10.71 | .113** | 0.052 |  |  |  |  |  |
| 4. Edu | 4.44 | 1.64 | 0.022 | -0.019 | .206** |  |  |  |  |
| 5. IRI-EC | 3.82 | 0.78 | .212** | .328** | .179** | -0.023 |  |  |  |
| 6. Pro_Int | 4.31 | 1.45 | .213** | .174** | 0.067 | .156** | .394** |  |  |
| 7. CRT | 0.52 | 0.40 | -.244** | -.250** | 0 | .141** | -.098* | -.109* |  |
| 8. IRI-PT | 3.68 | 0.66 | 0.019 | .141** | 0.04 | -0.02 | .486** | .292** | -0.058 |
| ** Correlation is significant at the 0.01 level (2-tailed). | | | | | |  |  |  |  |
| * Correlation is significant at the 0.05 level (2-tailed). | | | | | |  |  |  |  |

**Table 8.** Linear regression analysis predicting belief with gender, age, and education, empathic concern (IRI-EC), prosocial intentions (Prosocial), analytic reasoning (CRT), and perspective taking (IRI-PT). N =527.

| **Model** |  | **B** | ***S.E.*** | **β** | ***t*** | ***p*** | **R2Δ** | ***FΔ*** | **F sig** | ***Cohens f2*** |
| --- | --- | --- | --- | --- | --- | --- | --- | --- | --- | --- |
| 1 | Intercept | 2.935 | 0.505 |  | 5.812 | 0.000 |  |  |  |  |
|  | Gender | 0.6 | 0.213 | 0.122 | 2.814 | 0.005 |  |  |  |  |
|  | Age | 0.025 | 0.01 | 0.106 | 2.411 | 0.016 |  |  |  |  |
|  | Edu | 0.004 | 0.066 | 0.003 | 0.062 | 0.951 |  |  |  |  |
|  |  |  |  |  |  |  | 0.028 | 4.942 | 0.002 | 0.029 |
| 2 | Intercept | 1.146 | 0.641 |  | 1.789 | 0.074 |  |  |  |  |
|  | Gender | 0.282 | 0.221 | 0.057 | 1.279 | 0.202 |  |  |  |  |
|  | Age | 0.019 | 0.01 | 0.082 | 1.869 | 0.062 |  |  |  |  |
|  | Edu | -0.022 | 0.066 | -0.015 | -0.334 | 0.739 |  |  |  |  |
|  | IRI-EC | 0.374 | 0.155 | 0.118 | 2.408 | 0.016 |  |  |  |  |
|  | Prosocial | 0.262 | 0.08 | 0.154 | 3.286 | 0.001 |  |  |  |  |
|  |  |  |  |  |  |  | 0.046 | 13.028 | 0.000 | 0.05 |
| 3 | Intercept | 2.119 | 0.655 |  | 3.235 | 0.001 |  |  |  |  |
|  | Gender | 0.026 | 0.222 | 0.005 | 0.117 | 0.907 |  |  |  |  |
|  | Age | 0.018 | 0.01 | 0.078 | 1.822 | 0.069 |  |  |  |  |
|  | Edu | 0.03 | 0.065 | 0.02 | 0.452 | 0.651 |  |  |  |  |
|  | IRI-EC | 0.392 | 0.152 | 0.123 | 2.582 | 0.01 |  |  |  |  |
|  | Prosocial | 0.224 | 0.078 | 0.131 | 2.863 | 0.004 |  |  |  |  |
|  | CRT | -1.334 | 0.263 | -0.219 | -5.067 | 0.000 |  |  |  |  |
|  |  |  |  |  |  |  | 0.044 | 25.673 | 0.000 | 0.049 |
| 4 | Intercept | 3.159 | 0.756 |  | 4.178 | 0.000 |  |  |  |  |
|  | Gender | 0.008 | 0.22 | 0.002 | 0.036 | 0.971 |  |  |  |  |
|  | Age | 0.017 | 0.01 | 0.073 | 1.701 | 0.09 |  |  |  |  |
|  | Edu | 0.026 | 0.065 | 0.017 | 0.395 | 0.693 |  |  |  |  |
|  | IRI-EC | 0.577 | 0.166 | 0.181 | 3.482 | 0.001 |  |  |  |  |
|  | Prosocial | 0.251 | 0.078 | 0.147 | 3.201 | 0.001 |  |  |  |  |
|  | CRT | -1.338 | 0.262 | -0.219 | -5.111 | 0.000 |  |  |  |  |
|  | IRI-PT | -0.482 | 0.178 | -0.128 | -2.704 | 0.007 |  |  |  |  |
|  |  |  |  |  |  |  | 0.012 | 7.313 | 0.007 | 0.015 |

**Study 5**

Participants

Participants were drawn from across the undergraduate population of Case Western Reserve University and participated in a comprehensive study of social cognition involving two laboratory sessions of up to three hours, separated by a number of online surveys that participants completed on their own time. Payment for participation was variable, depending partly on performance. Only participants who completed all the measures of interest here are included in the analysis, of which there were 69 (33 female; average age 242.19 months, SD = 14.17).

Procedure and measures

Participants completed computerized (E-prime 2.0) versions of a number of well-validated mentalizing measures: Social Stories Questionnaire [8], Imposing Memory Task [9], DANVA facial emotion recognition task [10] the Reading the Mind in the Eyes test revised version [11] and the Interpersonal Perception Task [12]. Computerized laboratory versions of the Cognitive Reflections Test (CRT) and Intuitive Physics Task (IPT) were also administered.

The empathic concern (IRI-EC) was completed online through SurveyMonkey, and had good internal consistency, Cronbach’s α = 0.81. The peer-rated measure of empathy was obtained by asking participants to volunteer the email addresses of friends (both from university and high school) and their first-year roommate. Nominated peers were then emailed and asked to fill out a confidential survey about the participant, which included a number of subscales from the Emotional and Social Competency Inventory – University Edition (ESCI-U; [13]. Ratings from multiple peers were averaged. Of specific interest here was the Empathy subscale, which consists of 6 items including “Understands others from different backgrounds” and “Understands others by putting self into others shoes”. Items were scored on a 7-point Likert scale (1 = *Never*, 7 = *Always*). This scale had good internal consistency: Cronbach’s α = 0.84. Participants also completed the Autism Quotient measure (Cronbach α = 0.73).

Demographic variables

Sex, (Males = 1, Females = 2) and age in years (range: 18-23) were assessed and used as control variables.

Results and discussion

**Table 9.** Mean (M), standard deviations (SD), and bivariate correlations among study variables. N=69. Gender, 1 = *male*, 2 = *female*. Empathic concern (IRI-EC), Peer-rated empathy (peer-emp), analytic reasoning (CRT), mechanistic reasoning (IPT), Social Stories Questionnaire, Imposing Memory Task, Interpersonal Perception Task, Mind in the Eyes, DANVA, AQ (Autism Quotient).

| **Variable** | **Mean** | **S.D.** | **1** | **2** | **3** | **4** | **5** | **6** | **7** | **8** | **9** | **10** | **11** | **12** |
| --- | --- | --- | --- | --- | --- | --- | --- | --- | --- | --- | --- | --- | --- | --- |
| 1. Gender | 0.45 | 0.50 |  |  |  |  |  |  |  |  |  |  |  |  |
| 2. Age (months) | 242.19 | 14.03 | 0.14 |  |  |  |  |  |  |  |  |  |  |  |
| 3. Belief | 2.41 | 5.08 | -0.194 | -0.139 |  |  |  |  |  |  |  |  |  |  |
| 4. IRI-EC | 18.71 | 4.40 | -.407** | -0.111 | .425** |  |  |  |  |  |  |  |  |  |
| 5. Peer-Emp | 5.38 | 0.70 | -.309** | -0.065 | .369** | .287* |  |  |  |  |  |  |  |  |
| 6. CRT | 50.27 | 36.40 | .249* | 0.027 | -0.229 | -0.156 | -0.11 |  |  |  |  |  |  |  |
| 7. IPT | 63.22 | 15.81 | .399** | 0.227 | -0.222 | -.267* | -0.113 | .336** |  |  |  |  |  |  |
| 8. Social Stories | 64.01 | 10.76 | -0.169 | -0.053 | -0.092 | .258* | 0.013 | 0.074 | 0.093 |  |  |  |  |  |
| 9. Imposing Memory task | 81.06 | 5.72 | -0.084 | -0.065 | -0.016 | 0.035 | 0.077 | .319** | 0.143 | 0.205 |  |  |  |  |
| 10. Interpersonal Perception Task | 65.62 | 13.01 | .269* | 0.011 | -0.041 | -0.027 | -.324** | -0.029 | 0.125 | -0.102 | -0.041 |  |  |  |
| 11. Mind in the Eyes | 26.86 | 3.68 | -0.227 | 0.11 | 0.037 | 0.115 | 0.166 | 0.069 | 0.016 | 0.196 | 0.007 | -0.13 |  |  |
| 12. DANVA | 0.81 | 0.06 | -0.202 | 0.051 | 0.094 | -0.053 | 0.098 | 0.026 | 0.202 | -0.036 | 0.215 | -0.104 | 0.053 |  |
| 13. AQ | 14.94 | 5.71 | 0.179 | 0.141 | -.402** | -.439** | -.332** | 0.233 | 0.157 | -0.076 | -0.076 | -0.21 | 0.116 | -0.068 |
| ** Correlation is significant at the 0.01 level (2-tailed). | | | | | |  |  |  |  |  |  |  |  |  |
| * Correlation is significant at the 0.05 level (2-tailed). | | | | |  |  |  |  |  |  |  |  |  |  |

**Table 10.** Four-step hierarchical regression analysis predicting belief with gender, age, education, empathic concern (IRI-EC), Peer-rated empathy (peer-emp), analytic reasoning (CRT), mechanistic reasoning (IPT), Social Stories Questionnaire, Imposing Memory Task, Interpersonal Perception Task, Mind in the Eyes, DANVA, AQ (Autism Quotient).

| **Model** |  | **B** | ***S.E.*** | **β** | ***t*** | ***p*** | **R2Δ** | ***FΔ*** | **F sig** | ***Cohens f2*** |
| --- | --- | --- | --- | --- | --- | --- | --- | --- | --- | --- |
| 1 | Intercept | 13.24 | 10.582 |  | 1.251 | 0.215 |  |  |  |  |
|  | Gender | -1.804 | 1.229 | -0.178 | -1.468 | 0.147 |  |  |  |  |
|  | Age (months) | -0.041 | 0.044 | -0.114 | -0.943 | 0.349 |  |  |  |  |
|  |  |  |  |  |  |  | 0.05 | 1.751 | 0.182 | 0.053 |
| 2 | Intercept | -8.495 | 10.937 |  | -0.777 | 0.44 |  |  |  |  |
|  | Gender | 0.496 | 1.233 | 0.049 | 0.402 | 0.689 |  |  |  |  |
|  | Age (months) | -0.032 | 0.04 | -0.088 | -0.811 | 0.42 |  |  |  |  |
|  | IRI-EC | 0.412 | 0.139 | 0.356 | 2.96 | 0.004 |  |  |  |  |
|  | Peer_empathy | 1.996 | 0.833 | 0.276 | 2.396 | 0.02 |  |  |  |  |
|  |  |  |  |  |  |  | 0.206 | 8.857 | 0.000 | 0.277 |
| 3 | Intercept | -6.734 | 10.945 |  | -0.615 | 0.541 |  |  |  |  |
|  | Gender | 1.081 | 1.303 | 0.107 | 0.83 | 0.41 |  |  |  |  |
|  | Age (months) | -0.028 | 0.04 | -0.077 | -0.697 | 0.489 |  |  |  |  |
|  | IRI-EC | 0.391 | 0.14 | 0.338 | 2.799 | 0.007 |  |  |  |  |
|  | Peer_empathy | 1.989 | 0.831 | 0.276 | 2.395 | 0.02 |  |  |  |  |
|  | CRT | -0.02 | 0.016 | -0.144 | -1.251 | 0.216 |  |  |  |  |
|  | IPT | -0.025 | 0.04 | -0.078 | -0.623 | 0.535 |  |  |  |  |
|  |  |  |  |  |  |  | 0.029 | 1.278 | 0.286 | 0.042 |
| 4 | Intercept | -5.619 | 17.414 |  | -0.323 | 0.748 |  |  |  |  |
|  | Gender | 1.225 | 1.46 | 0.121 | 0.839 | 0.405 |  |  |  |  |
|  | Age (months) | -0.029 | 0.041 | -0.08 | -0.706 | 0.483 |  |  |  |  |
|  | IRI-EC | 0.388 | 0.157 | 0.335 | 2.467 | 0.017 |  |  |  |  |
|  | Peer_empathy | 1.417 | 0.953 | 0.196 | 1.487 | 0.143 |  |  |  |  |
|  | CRT | -0.015 | 0.018 | -0.11 | -0.875 | 0.385 |  |  |  |  |
|  | IPT | -0.026 | 0.043 | -0.081 | -0.596 | 0.554 |  |  |  |  |
|  | Social_Stories | -0.082 | 0.057 | -0.173 | -1.436 | 0.157 |  |  |  |  |
|  | ImposingMemory | 0.004 | 0.108 | 0.004 | 0.037 | 0.971 |  |  |  |  |
|  | Inter_Perc_Task | -0.011 | 0.05 | -0.029 | -0.226 | 0.822 |  |  |  |  |
|  | Mind in Eyes | 0.077 | 0.163 | 0.056 | 0.474 | 0.638 |  |  |  |  |
|  | DANVA | 9.94 | 10.511 | 0.114 | 0.946 | 0.348 |  |  |  |  |
|  | AQ | -0.16 | 0.124 | -0.18 | -1.287 | 0.204 |  |  |  |  |
|  |  |  |  |  |  |  | 0.062 | 0.892 | 0.507 | 0.095 |

**Study 6**

Participants

Six hundred and thirty-seven participants were recruited through Amazon’s Mechanical Turk, where they were linked to a survey hosted by SurveyMonkey. Participants were required to have completed 500 HITs on Mechanical Turk with an approval rating of 95% or higher. There was no geographical restriction for participation. One hundred and seventy-eight people were excluded from the final analyses for failing to answer our catch-questions correctly, leaving a total of 527 participants with complete data (253 females, 55.1% females; average age 36.23, SD = 12.97). Participants were paid $0.75 for their HIT.

Procedure and measures

Participants first completed the CRT, followed by the empathic concern (IRI-EC) and perspective taking (IRI-PT) of the Interpersonal Reactivity Index. Both the IRI-EC and IRI-PT had good internal consistency (Cronbach’s α = 0.86) and (Cronbach’s α = 0.84), respectively.

Participants then completed the Intuitive Physics Test (IPT) followed by the empathizing quotient (EQ) and systemizing quotient (SQ) [14]. The EQ had acceptable internal consistency (Cronbach’s α = 0.72), but the SQ did not (Cronbach’s α = 0.30), and so it was dropped from subsequent analyses. Lastly, participants completed the single-item measure of belief followed by demographic questions. The survey included additional measures which are not of relevance here.

Demographic variables

Sex, (Males = 1, Females = 2), age in years (range: 19-75), and level of education [1 = some high school or less(1.1%), 2 = high school diploma (11.1%), 3 = some college (21.8%), 4 = associates/2-year degree (10.2%), 5 = bachelor’s/4-year degree (33.1%), 6 = some graduate school (4.4%), 7 = master’s degree (14.8%), 8 = doctorate degree (3.5 %), 0 = other (0.0%)] were also assessed and used as control variables.

Results and discussion

**Table 11.** Mean (M), standard deviations (SD), and bivariate correlations among study variables. N=459. Gender, 1 = *male*, 2 = *female*. Empathic concern (IRI-EC), analytic reasoning (CRT), mechanistic reasoning (IPT), perspective taking (IRI-PT), empathizing quotient (EQ).

| **Variable** | **Mean** | **S.D.** | **1** | **2** | **3** | **4** | **5** | **6** | **7** | **8** |
| --- | --- | --- | --- | --- | --- | --- | --- | --- | --- | --- |
| 1. Gender | 1.55 | 0.50 |  |  |  |  |  |  |  |  |
| 2. Age | 36.23 | 12.97 | 0.05 |  |  |  |  |  |  |  |
| 3. Edu | 4.53 | 1.70 | -0.03 | 0.003 |  |  |  |  |  |  |
| 4. Belief | 5.16 | 2.39 | 0.06 | 0.084 | 0.005 |  |  |  |  |  |
| 5. IRI-EC | 3.78 | 0.75 | .233** | .149** | -0.05 | .209** |  |  |  |  |
| 6. CRT | 0.51 | 0.41 | -.260** | 0.045 | .148** | -.159** | -0.084 |  |  |  |
| 7. IPT | 8.22 | 2.70 | -.215** | 0.056 | -0.021 | -.154** | -0.064 | .350** |  |  |
| 8. IRI-PT | 3.59 | 0.70 | .099* | 0.043 | 0.046 | 0.068 | .529** | 0.001 | -0.075 |  |
| 9. EQ | 22.43 | 5.81 | 0.064 | -0.091 | 0.087 | .163** | .386** | -.150** | -.140** | .346** |
| ** Correlation is significant at the 0.01 level (2-tailed). | | | | | |  |  |  |  |  |
| * Correlation is significant at the 0.05 level (2-tailed). | | | | | |  |  |  |  |  |

**Table 11.** Linear regression analysis predicting belief with gender, age, education, empathic concern (IRI-EC), analytic reasoning (CRT), mechanistic reasoning (IPT), perspective taking (IRI-PT), and empathizing quotient (EQ).

| **Model** |  | **B** | ***S.E.*** | **β** | ***t*** | ***p*** | **R2Δ** | ***FΔ*** | **F sig** | ***Cohens f2*** |
| --- | --- | --- | --- | --- | --- | --- | --- | --- | --- | --- |
| 1 | Intercept | 4.202 | 0.559 |  | 7.511 | 0 |  |  |  |  |
|  | Gender | 0.244 | 0.224 | 0.051 | 1.09 | 0.276 |  |  |  |  |
|  | Age | 0.015 | 0.009 | 0.081 | 1.74 | 0.083 |  |  |  |  |
|  | Edu | 0.008 | 0.066 | 0.006 | 0.122 | 0.903 |  |  |  |  |
|  |  |  |  |  |  |  | 0.01 | 1.472 | 0.221 | 0.01 |
| 2 | Intercept | 2.254 | 0.719 |  | 3.134 | 0.002 |  |  |  |  |
|  | Gender | 0.028 | 0.226 | 0.006 | 0.126 | 0.9 |  |  |  |  |
|  | Age | 0.01 | 0.009 | 0.054 | 1.156 | 0.248 |  |  |  |  |
|  | Edu | 0.021 | 0.065 | 0.015 | 0.318 | 0.75 |  |  |  |  |
|  | IRI-EC | 0.638 | 0.152 | 0.2 | 4.195 | 0.000 |  |  |  |  |
|  |  |  |  |  |  |  | 0.037 | 17.598 | 0.000 | 0.039 |
| 3 | Intercept | 3.731 | 0.821 |  | 4.545 | 0 |  |  |  |  |
|  | Gender | -0.236 | 0.232 | -0.049 | -1.02 | 0.308 |  |  |  |  |
|  | Age | 0.013 | 0.008 | 0.069 | 1.509 | 0.132 |  |  |  |  |
|  | Edu | 0.04 | 0.064 | 0.029 | 0.627 | 0.531 |  |  |  |  |
|  | IRI-EC | 0.618 | 0.15 | 0.194 | 4.127 | 0.000 |  |  |  |  |
|  | CRT | -0.724 | 0.291 | -0.124 | -2.49 | 0.013 |  |  |  |  |
|  | IPT | -0.099 | 0.043 | -0.112 | -2.295 | 0.022 |  |  |  |  |
|  |  |  |  |  |  |  | 0.034 | 8.38 | 0.000 | 0.037 |
| 4 | Intercept | 3.668 | 0.911 |  | 4.028 | 0.000 |  |  |  |  |
|  | Gender | -0.215 | 0.232 | -0.045 | -0.929 | 0.354 |  |  |  |  |
|  | Age | 0.014 | 0.009 | 0.079 | 1.704 | 0.089 |  |  |  |  |
|  | Edu | 0.032 | 0.065 | 0.023 | 0.492 | 0.623 |  |  |  |  |
|  | IRI-EC | 0.633 | 0.183 | 0.198 | 3.453 | 0.001 |  |  |  |  |
|  | CRT | -0.639 | 0.293 | -0.11 | -2.18 | 0.03 |  |  |  |  |
|  | IPT | -0.097 | 0.043 | -0.11 | -2.245 | 0.025 |  |  |  |  |
|  | IRI-PT | -0.26 | 0.184 | -0.076 | -1.41 | 0.159 |  |  |  |  |
|  | EQ | 0.037 | 0.021 | 0.089 | 1.734 | 0.084 |  |  |  |  |
|  |  |  |  |  |  |  | 0.009 | 2.142 | 0.119 | 0.009 |

**Study 7.**

Participants

Three hundred and eighteen participants were recruited through Amazon’s Mechanical Turk, where they were linked to a survey hosted by SurveyMonkey. Participants were required to have completed 50 HITs on Mechanical Turk with an approval rating of 95% or higher. Location of participants was not limited. One hundred and sixty-two people were excluded from the final analyses for failing to answer our catch-questions correctly, leaving a total of 156 participants with complete data (64 females, 41.0% females; average age 30.51, SD = 9.02). Participants were paid $0.45 for their HIT.

Procedure and measures

Participants first answered demographic questions, which were followed by the empathic concern subscale (IRI-EC;Cronbach’s α = 0.68). Participants completed the 20-item UCLA Loneliness Scale, in which questions are answered on a Likert scale with responses ranging from 1 (*Never*) to 4 (*Often*) [15]. This scale had good internal consistency: Cronbach’s α = 0.93. Participants then filled out the DASS-21, which consists of three 7-item subscales assessing depression, anxiety, and stress [16]. Questions are answered on a Likert-scale with responses ranging from 0 (*Did not apply to me at all*) to 3 (*Applied to me very much, or most of the time*). The depression subscale had good internal consistency: Cronbach’s α = 0.88. Finally, participants completed the Quick Inventory of Depressive Symptomology 16-item Self-Report (QIDS-SR_16_), which assesses changes in sleep schedule, weight/appetite, negative thoughts, and energy level [17]. This scale had good internal consistency: Cronbach’s α = 0.79.

The survey included additional measures which are not of relevance here.

Demographic variables

Sex, (Males = 1, Females = 2), age in years (range: 18-62), and level of education [1 = some high school or less(3.8%), 2 = high school diploma (7.7%), 3 = some college (17.9%), 4 = associates/2-year degree (3.2%), 5 = bachelor’s/4-year degree (37.2%), 6 = some graduate school (2.6%), 7 = master’s degree (24.4%), 8 = doctorate degree (2.6%), 0 = other (0.6%)] were also assessed and used as control variables.

Results and discussion

**Table 13.** Mean (M), standard deviations (SD), and bivariate correlations among study variables. N=155. Gender, *1* = *male*, 2 = *female*. Empathic concern (IRI-EC), Loneliness, Depression and Anxiety (QIDS).

| **Variable** | **Mean** | **S.D.** | **1** | **2** | **3** | **4** | **5** | **6** | **7** |
| --- | --- | --- | --- | --- | --- | --- | --- | --- | --- |
| 1. Gender | 1.41 | 0.49 |  |  |  |  |  |  |  |
| 2. Age | 30.51 | 9.02 | 0.149 |  |  |  |  |  |  |
| 3. Edu | 4.78 | 1.85 | 0.035 | 0.133 |  |  |  |  |  |
| 4. Belief | 5.08 | 2.34 | 0.11 | .183* | .165* |  |  |  |  |
| 5. IRI-EC | 3.74 | 0.59 | .201* | .213** | .182* | .318** |  |  |  |
| 6. Loneliness | 39.67 | 12.31 | 0.057 | -.233** | -.237** | -.259** | -.298** |  |  |
| 7. Depression | 12.92 | 4.86 | 0.005 | -.308** | -0.146 | -.203* | -.198* | .741** |  |
| 8. QIDS | 5.81 | 4.27 | -0.011 | -0.142 | -.253** | -0.137 | -0.143 | .539** | .614** |
| * Correlation is significant at the 0.05 level (2-tailed). | | | | | |  |  |  |  |
| ** Correlation is significant at the 0.01 level (2-tailed). | | | | | |  |  |  |  |

**Table 14.** Linear regression predicting belief with gender, age, education, empathic concern (IRI-EC), Loneliness, Depression (DASS) and Depressive Symptomatology (QIDS).

| **Model** |  | **B** | ***S.E.*** | **β** | ***t*** | ***p*** | **R2Δ** | ***FΔ*** | **F sig** | ***Cohens f2*** |
| --- | --- | --- | --- | --- | --- | --- | --- | --- | --- | --- |
| 1 | Intercept | 2.482 | 0.876 |  | 2.833 | 0.005 |  |  |  |  |
|  | Gender | 0.385 | 0.379 | 0.081 | 1.015 | 0.312 |  |  |  |  |
|  | Age | 0.039 | 0.021 | 0.152 | 1.888 | 0.061 |  |  |  |  |
|  | Edu | 0.18 | 0.101 | 0.142 | 1.784 | 0.076 |  |  |  |  |
|  |  |  |  |  |  |  | 0.06 | 3.218 | 0.025 | 0.064 |
| 2 | Intercept | -0.613 | 1.252 |  | -0.49 | 0.625 |  |  |  |  |
|  | Gender | 0.167 | 0.372 | 0.035 | 0.447 | 0.655 |  |  |  |  |
|  | Age | 0.028 | 0.02 | 0.107 | 1.352 | 0.178 |  |  |  |  |
|  | Edu | 0.128 | 0.099 | 0.101 | 1.292 | 0.198 |  |  |  |  |
|  | IRI-EC | 1.073 | 0.32 | 0.27 | 3.357 | 0.001 |  |  |  |  |
|  |  |  |  |  |  |  | 0.066 | 11.268 | 0.001 | 0.076 |
| 3 | Intercept | 1.456 | 1.659 |  | 0.878 | 0.381 |  |  |  |  |
|  | Gender | 0.28 | 0.377 | 0.059 | 0.743 | 0.459 |  |  |  |  |
|  | Age | 0.019 | 0.021 | 0.075 | 0.906 | 0.366 |  |  |  |  |
|  | Edu | 0.097 | 0.103 | 0.076 | 0.942 | 0.348 |  |  |  |  |
|  | IRI-EC | 0.912 | 0.332 | 0.229 | 2.75 | 0.007 |  |  |  |  |
|  | Loneliness | -0.029 | 0.023 | -0.152 | -1.25 | 0.213 |  |  |  |  |
|  | Depression | -0.012 | 0.062 | -0.025 | -0.197 | 0.844 |  |  |  |  |
|  | QIDS | 0.013 | 0.055 | 0.024 | 0.239 | 0.812 |  |  |  |  |
|  |  |  |  |  |  |  | 0.021 | 1.207 | 0.309 | 0.025 |

**Study 8**

Participants

Six hundred and one participants were recruited through Amazon’s Mechanical Turk, where they were linked to a survey hosted by SurveyMonkey. Participants were required to have completed 500 HITs on Mechanical Turk with an approval rating of 97% or higher. There was not a geographical restriction for participation. Two hundred and thirty people were excluded from the final analyses for failing to answer our catch-questions correctly, leaving a total of 371 participants with complete data (210 females, 43.1% females; average age 35.44, SD = 12.18). Participants were paid $0.50 for their HIT.

Procedure and measures

Participants were first presented with the CRT and then IRI-EC (Cronbach’s α = .85), followed by the 33-item Marlowe-Crowne Social Desirability Scale (MCSD) [18] 1960). The MCSD is completed with True or False responses and is comprised of two factors, Attribution and Denial. The Attribution factor addresses the tendency to exacerbate self-ascriptions of socially desirable characteristics; the Denial factor addresses the tendency to deny socially undesirable self-ascriptions. Answering ‘True’ to the attribution items indicates socially desirable responses (i.e. *I’m always willing to admit it when I make a mistake*) whereas answering ‘False” indicates socially desirable responses for the denial items (i.e. *At times I have really insisted on having things my own way.)* Higher scores on each factor reflect higher social desirability. Both factors had acceptable internal consistency: Attribution, Cronbach’s α = .80; Denial, Cronbach’s α = .80.

Participants were then presented with the single-item measure assessing belief. This was followed by two single-item questions designed to address whether frequency of social religious practices (*How often do you attend religious services, pray or meditate with others?***)** or attendance at social events associated with their religious affiliation (*How often do you attend social events organized on behalf of your church or religious group?*) Both items were answered with a 6-point scale (1 = Never; 2 = Once or more per year; 3 = Once or more per month; 4 = Weekly; 5 = More than once a week; 6 = Daily). Finally, participants answered the same demographic questions as the previous studies.

Demographic variables

Sex, (Males = 1, Females = 2), age in years (range: 19-69), and level of education [1 = some high school or less (0.5%), 2 = high school diploma (6.5%), 3 = some college (18.9%), 4 = associates/2-year degree (7.8%), 5 = bachelor’s/4-year degree (36.1%), 6 = some graduate school (4.3%), 7 = master’s degree (24.5%), 8 = doctorate degree (1.1%), 0 = other (0.3%)] were also assessed and used as control variables.

Results and discussion

**Table 15.** Mean (M), standard deviations (SD), and bivariate correlations among study variables. N=370. Gender, *1* = *male*, 2 = *female*. Empathic concern (IRI-EC), analytic reasoning (CRT), attributing socially desirable responses (Attribution), denying socially undesirable responses (Denial), and two items on attendance of religious and spiritual practices (att/prayer/meditation) and social benefits on behalf of one’s religious group (Social_events)

| **Variable** | **Mean** | **S.D.** | **1** | **2** | **3** | **4** | **5** | **6** | **7** | **8** | **9** |
| --- | --- | --- | --- | --- | --- | --- | --- | --- | --- | --- | --- |
| 1.Gender | 1.57 | 0.50 |  |  |  |  |  |  |  |  |  |
| 2. Age | 35.44 | 12.19 | -0.063 |  |  |  |  |  |  |  |  |
| 3. Edu | 4.89 | 1.62 | 0.048 | -0.016 |  |  |  |  |  |  |  |
| 4. Belief | 5.28 | 2.24 | -.130* | 0.038 | -0.013 |  |  |  |  |  |  |
| 5. IRI-EC | 3.79 | 0.74 | -.205** | 0.07 | -0.031 | .302** |  |  |  |  |  |
| 6. CRT | 0.56 | 0.41 | 0.097 | 0.085 | 0.088 | -.167** | -0.1 |  |  |  |  |
| 7. Attribution | 11.25 | 3.86 | 0.02 | -.162** | .144** | .255** | .297** | -.216** |  |  |  |
| 8. Denial | 6.46 | 3.72 | 0.017 | 0.094 | 0.013 | .194** | .273** | -0.074 | .533** |  |  |
| 9. att/prayer/meditation | 2.88 | 1.71 | 0.034 | -.111* | .173** | .540** | .209** | -.176** | .440** | .279** |  |
| 10. Social_events | 2.15 | 1.30 | 0.058 | -.188** | .181** | .439** | .183** | -.185** | .461** | .261** | .774** |
| * Correlation is significant at the 0.05 level (2-tailed). | | | | | |  |  |  |  |  |  |
| ** Correlation is significant at the 0.01 level (2-tailed). | | | | | |  |  |  |  |  |  |

**Table 16.** Linear regression analysis predicting belief with gender, age, education, empathic concern (IRI-EC), analytic reasoning (CRT), attributing socially desirable responses (Attribution), denying socially undesirable responses (Denial), and two items on attendance of religious and spiritual practices (att/pray/med) and social benefits on behalf of one’s religious group (SocialEvents)

| **Model** |  | **B** | ***S.E.*** | **β** | ***t*** | ***p*** | **R2Δ** | ***FΔ*** | **F sig** | ***Cohens f2*** |
| --- | --- | --- | --- | --- | --- | --- | --- | --- | --- | --- |
| 1 | Intercept | 6.031 | 0.628 |  | 9.609 | 0 |  |  |  |  |
|  | Gender | -0.579 | 0.235 | -0.128 | -2.463 | 0.014 |  |  |  |  |
|  | Age | 0.006 | 0.01 | 0.03 | 0.58 | 0.562 |  |  |  |  |
|  | Edu | -0.009 | 0.072 | -0.006 | -0.119 | 0.905 |  |  |  |  |
|  |  |  |  |  |  |  | 0.018 | 2.224 | 0.085 | 0.018 |
| 2 | Intercept | 2.398 | 0.883 |  | 2.716 | 0.007 |  |  |  |  |
|  | Gender | -0.319 | 0.23 | -0.071 | -1.386 | 0.167 |  |  |  |  |
|  | Age | 0.003 | 0.009 | 0.014 | 0.278 | 0.782 |  |  |  |  |
|  | Edu | 0 | 0.069 | 0 | -0.004 | 0.997 |  |  |  |  |
|  | IRI-EC | 0.869 | 0.154 | 0.287 | 5.636 | 0.000 |  |  |  |  |
|  |  |  |  |  |  |  | 0.079 | 31.764 | 0.000 | 0.087 |
| 3 | Intercept | 2.712 | 0.883 |  | 3.073 | 0.002 |  |  |  |  |
|  | Gender | -0.269 | 0.229 | -0.059 | -1.174 | 0.241 |  |  |  |  |
|  | Age | 0.005 | 0.009 | 0.027 | 0.546 | 0.586 |  |  |  |  |
|  | Edu | 0.015 | 0.069 | 0.011 | 0.225 | 0.822 |  |  |  |  |
|  | IRI-EC | 0.833 | 0.153 | 0.275 | 5.43 | 0.000 |  |  |  |  |
|  | CRT | -0.754 | 0.277 | -0.137 | -2.726 | 0.007 |  |  |  |  |
|  |  |  |  |  |  |  | 0.018 | 7.433 | 0.007 | 0.02 |
| 4 | Intercept | 2.265 | 0.893 |  | 2.537 | 0.012 |  |  |  |  |
|  | Gender | -0.345 | 0.227 | -0.076 | -1.52 | 0.129 |  |  |  |  |
|  | Age | 0.009 | 0.009 | 0.049 | 0.967 | 0.334 |  |  |  |  |
|  | Edu | -0.022 | 0.069 | -0.016 | -0.325 | 0.746 |  |  |  |  |
|  | IRI-EC | 0.648 | 0.161 | 0.214 | 4.032 | 0.000 |  |  |  |  |
|  | CRT | -0.569 | 0.279 | -0.103 | -2.035 | 0.043 |  |  |  |  |
|  | Attribution | 0.093 | 0.037 | 0.16 | 2.544 | 0.011 |  |  |  |  |
|  | Denial | 0.024 | 0.036 | 0.04 | 0.665 | 0.507 |  |  |  |  |
|  |  |  |  |  |  |  | 0.028 | 5.911 | 0.003 | 0.032 |
| 5 | Intercept | 2.393 | 0.774 |  | 3.093 | 0.002 |  |  |  |  |
|  | Gender | -0.468 | 0.197 | -0.104 | -2.374 | 0.018 |  |  |  |  |
|  | Age | 0.016 | 0.008 | 0.089 | 1.991 | 0.047 |  |  |  |  |
|  | Edu | -0.127 | 0.061 | -0.091 | -2.09 | 0.037 |  |  |  |  |
|  | IRI-EC | 0.482 | 0.14 | 0.159 | 3.442 | 0.001 |  |  |  |  |
|  | CRT | -0.252 | 0.244 | -0.046 | -1.035 | 0.301 |  |  |  |  |
|  | Attribution | -0.01 | 0.033 | -0.017 | -0.29 | 0.772 |  |  |  |  |
|  | Denial | -0.002 | 0.031 | -0.003 | -0.05 | 0.96 |  |  |  |  |
|  | att/pray/med | 0.619 | 0.089 | 0.472 | 6.949 | 0.000 |  |  |  |  |
|  | SocialEvents | 0.144 | 0.119 | 0.084 | 1.209 | 0.228 |  |  |  |  |
|  |  |  |  |  |  |  | 0.218 | 61.345 | 0.000 | 0.341 |

1. Baron-Cohen, S., et al., *Are intuitive physics and intuitive psychology independent? A test with children with Asperger Syndrome.* Journal of Developmental and Learning Disorders, 2001. **5**(1): p. 47-78.

2. Davis, M.H., *Measuring individual differences in empathy: evidence for a multidimensional approach.* Journal of personality and social psychology, 1983. **44**(1): p. 113.

3. Frederick, S., *Cognitive reflection and decision making.* Journal of Economic perspectives, 2005: p. 25-42.

4. Raven, J.C. and J.B. Walshaw, *Vocabulary Tests.* British Journal of Medical Psychology, 1944. **20**: p. 185-194.

5. McFarland, S., M. Webb, and D. Brown, *All humanity is my ingroup: A measure and studies of identification with all humanity.* Journal of Personality and Social Psychology, 2012. **103**(5): p. 830.

6. Iyer, R., et al., *Understanding Libertarian Morality: The Psychological Dispositions of Self-Identified Libertarians.* Plos One, 2012. **7**(8).

7. Pavey, L., T. Greitemeyer, and P. Sparks, *Highlighting relatedness promotes prosocial motives and behavior.* Pers Soc Psychol Bull, 2011. **37**(7): p. 905-17.

8. Lawson, E.T., Baron-Cohen, S & Wheelwright, S., *Empathising and systemising in adults with and without Aspberger syndrome.* Journal of autism and developmental disorders, 2004. **34**(3): p. 301-310.

9. Kinderman, P., Dunbar, R., & Bentall, R.P., *Theory-of-mind deficits and causal attributions.* British Journal of Psychology, 1998. **89**(2): p. 191-204.

10. Nowicki, S. and M.P. Duke, *Nonverbal receptivity: The Diagnostic Analysis of Nonverbal Accuracy (DANVA).* 2001.

11. Baron‐Cohen, S., et al., *The “Reading the Mind in the Eyes” test revised version: A study with normal adults, and adults with Asperger syndrome or high‐functioning autism.* Journal of child psychology and psychiatry, 2001. **42**(2): p. 241-251.

12. Costanzo, M. and D. Archer, *Interpreting the Expressive Behavior of Others - the Interpersonal Perception Task.* Journal of Nonverbal Behavior, 1989. **13**(4): p. 225-245.

13. Boyatzis, R.E. and D. Goleman, *Emotional and Social Competencies Inventory.* Boston: The Hay Group, 2007.

14. Baron-Cohen, S. and S. Wheelwright, *The empathy quotient: an investigation of adults with Asperger syndrome or high functioning autism, and normal sex differences.* Journal of autism and developmental disorders, 2004. **34**(2): p. 163-175.

15. Peplau, L.A. and C.E. Cutrona, *The revised UCLA Loneliness Scale: Concurrent and discriminant validity evidence.* Journal of personality and social psychology, 1980. **39**(3): p. 472-480.

16. Henry, J.D. and J.R. Crawford, *The short‐form version of the Depression Anxiety Stress Scales (DASS‐21): Construct validity and normative data in a large non‐clinical sample.* British Journal of Clinical Psychology, 2005. **44**(2): p. 227-239.

17. Trivedi, M.H., et al., *The Inventory of Depressive Symptomatology, Clinician Rating (IDS-C) and Self-Report (IDS-SR), and the Quick Inventory of Depressive Symptomatology, Clinician Rating (QIDS-C) and Self-Report (QIDS-SR) in public sector patients with mood disorders: a psychometric evaluation.* Psychological medicine, 2004. **34**(01): p. 73-82.

18. Crowne, D.P. and D. Marlowe, *A New Scale of Social Desirability Independent of Psychopathology.* Journal of Consulting Psychology, 1960. **24**(4): p. 349-354.
