# Supplementary material for: Correction: Why Do You Believe in God? Relationships between Religious Belief, Analytic Thinking, Mentalizing and Moral Concern
Source: PLoS One. 2016 May 11;11(5):e0155283. doi: 10.1371/journal.pone.0155283 (PMC4864305; doi:10.1371/journal.pone.0155283)
Supplement: S1 File — (DOCX) [file pone.0155283.s001.docx]

Demographic variables

Sex, (Females = 1, Males = 2) and age in years (range: 18-23) were assessed and used as control variables.

Results and discussion

**Table 9.** Mean (M), standard deviations (SD), and bivariate correlations among study variables. N=69. Gender, 1 = *male*, 2 = *female*. Empathic concern (IRI-EC), Peer-rated empathy (peer-emp), analytic reasoning (CRT), mechanistic reasoning (IPT), Social Stories Questionnaire, Imposing Memory Task, Interpersonal Perception Task, Mind in the Eyes, DANVA, AQ (Autism Quotient).

| **Variable** | **Mean** | **S.D.** | **1** | **2** | **3** | **4** | **5** | **6** | **7** | **8** | **9** | **10** | **11** | **12** |
| --- | --- | --- | --- | --- | --- | --- | --- | --- | --- | --- | --- | --- | --- | --- |
| 1. Gender | 0.45 | 0.50 |  |  |  |  |  |  |  |  |  |  |  |  |
| 2. Age (months) | 242.19 | 14.03 | 0.14 |  |  |  |  |  |  |  |  |  |  |  |
| 3. Belief | 2.41 | 5.08 | -0.194 | -0.139 |  |  |  |  |  |  |  |  |  |  |
| 4. IRI-EC | 18.71 | 4.40 | -.407** | -0.111 | .425** |  |  |  |  |  |  |  |  |  |
| 5. Peer-Emp | 5.38 | 0.70 | -.309** | -0.065 | .369** | .287* |  |  |  |  |  |  |  |  |
| 6. CRT | 50.27 | 36.40 | .249* | 0.027 | -0.229 | -0.156 | -0.11 |  |  |  |  |  |  |  |
| 7. IPT | 63.22 | 15.81 | .399** | 0.227 | -0.222 | -.267* | -0.113 | .336** |  |  |  |  |  |  |
| 8. Social Stories | 64.01 | 10.76 | -0.169 | -0.053 | -0.092 | .258* | 0.013 | 0.074 | 0.093 |  |  |  |  |  |
| 9. Imposing Memory task | 81.06 | 5.72 | -0.084 | -0.065 | -0.016 | 0.035 | 0.077 | .319** | 0.143 | 0.205 |  |  |  |  |
| 10. Interpersonal Perception Task | 65.62 | 13.01 | .269* | 0.011 | -0.041 | -0.027 | -.324** | -0.029 | 0.125 | -0.102 | -0.041 |  |  |  |
| 11. Mind in the Eyes | 26.86 | 3.68 | -0.227 | 0.11 | 0.037 | 0.115 | 0.166 | 0.069 | 0.016 | 0.196 | 0.007 | -0.13 |  |  |
| 12. DANVA | 0.81 | 0.06 | -0.202 | 0.051 | 0.094 | -0.053 | 0.098 | 0.026 | 0.202 | -0.036 | 0.215 | -0.104 | 0.053 |  |
| 13. AQ | 14.94 | 5.71 | 0.179 | 0.141 | -.402** | -.439** | -.332** | 0.233 | 0.157 | -0.076 | -0.076 | -0.21 | 0.116 | -0.068 |
| ** Correlation is significant at the 0.01 level (2-tailed). | | | | | |  |  |  |  |  |  |  |  |  |
| * Correlation is significant at the 0.05 level (2-tailed). | | | | |  |  |  |  |  |  |  |  |  |  |

Demographic variables

Sex, (Females = 1, Males = 2), age in years (range: 19-69), and level of education [1 = some high school or less (0.5%), 2 = high school diploma (6.5%), 3 = some college (18.9%), 4 = associates/2-year degree (7.8%), 5 = bachelor’s/4-year degree (36.1%), 6 = some graduate school (4.3%), 7 = master’s degree (24.5%), 8 = doctorate degree (1.1%), 0 = other (0.3%)] were also assessed and used as control variables.

Results and discussion

**Table 15.** Mean (M), standard deviations (SD), and bivariate correlations among study variables. N=370. Gender, *1* = *female*, 2 = *male*. Empathic concern (IRI-EC), analytic reasoning (CRT), attributing socially desirable responses (Attribution), denying socially undesirable responses (Denial), and two items on attendance of religious and spiritual practices (att/prayer/meditation) and social benefits on behalf of one’s religious group (Social_events)

| **Variable** | **Mean** | **S.D.** | **1** | **2** | **3** | **4** | **5** | **6** | **7** | **8** | **9** |
| --- | --- | --- | --- | --- | --- | --- | --- | --- | --- | --- | --- |
| 1.Gender | 1.43 | 0.50 |  |  |  |  |  |  |  |  |  |
| 2. Age | 35.44 | 12.19 | -0.063 |  |  |  |  |  |  |  |  |
| 3. Edu | 4.89 | 1.62 | 0.048 | -0.016 |  |  |  |  |  |  |  |
| 4. Belief | 5.28 | 2.24 | .130* | 0.038 | -0.013 |  |  |  |  |  |  |
| 5. IRI-EC | 3.79 | 0.74 | -.205** | 0.07 | -0.031 | .302** |  |  |  |  |  |
| 6. CRT | 0.56 | 0.41 | 0.097 | 0.085 | 0.088 | -.167** | -0.1 |  |  |  |  |
| 7. Attribution | 11.25 | 3.86 | 0.02 | -.162** | .144** | .255** | .297** | -.216** |  |  |  |
| 8. Denial | 6.46 | 3.72 | 0.017 | 0.094 | 0.013 | .194** | .273** | -0.074 | .533** |  |  |
| 9. att/prayer/meditation | 2.88 | 1.71 | 0.034 | -.111* | .173** | .540** | .209** | -.176** | .440** | .279** |  |
| 10. Social_events | 2.15 | 1.30 | 0.058 | -.188** | .181** | .439** | .183** | -.185** | .461** | .261** | .774** |
| * Correlation is significant at the 0.05 level (2-tailed). | | | | | |  |  |  |  |  |  |
| ** Correlation is significant at the 0.01 level (2-tailed). | | | | | |  |  |  |  |  |  |
